# Circulating lipid profiles and atrial fibrillation: from incident risk to post-ablation recurrence

**DOI:** 10.3389/fcvm.2026.1909014

**Published:** 2026-09-02

**Authors:** Mi Yan, Jingfeng Zou, Wen Peng

**Affiliations:** Department of General Practice, Union Hospital, Tongji Medical College, Huazhong University of Science and Technology, Wuhan, China

**Keywords:** atrial fibrillation, catheter ablation, cholesterol paradox, circulating lipids, insulin resistance, post-ablation recurrence

## Abstract

Circulating lipids are widely used in cardiovascular risk assessment, but their relationship with atrial fibrillation (AF) differs from the conventional atherosclerotic framework. Higher triglyceride levels have often been associated with increased AF risk, whereas lower total cholesterol, low-density lipoprotein cholesterol, or remnant cholesterol have paradoxically been linked to greater risk in some populations. These inconsistent findings suggest that lipid measures may reflect stage-specific metabolic and clinical phenotypes rather than uniform causal exposures. This narrative review compares lipid-related evidence across two clinically distinct outcomes: incident AF and recurrence after catheter ablation. We examine conventional lipid measures, apolipoproteins, lipoprotein(a), and composite or longitudinal indices related to insulin resistance and metabolic burden. For incident AF, cholesterol-related markers show inverse, nonlinear, and population-dependent associations, while Mendelian randomization studies have not consistently supported direct causal effects. Post-ablation recurrence represents a treatment-response outcome occurring in patients with an established arrhythmogenic substrate and may be influenced by pre-existing atrial remodeling, lesion formation, postprocedural inflammation and repair, and residual triggers. In this setting, several composite or longitudinal indices integrating glycemia, adiposity, inflammation, or temporal variation have shown more consistent associations than individual conventional lipid measures in some cohorts. However, the evidence remains predominantly observational and is limited by heterogeneity in study populations, ablation modalities, recurrence definitions, rhythm-monitoring strategies, covariate adjustment, and treatment background. Studies of lipid-lowering and cardiometabolic therapies further indicate that changes in lipid concentrations do not necessarily produce parallel changes in AF risk or post-ablation rhythm outcomes. Accordingly, circulating lipids and their derived indices may be better interpreted as stage-specific markers of systemic metabolic burden, inflammatory status, comorbidity, and atrial substrate rather than as isolated causal factors or direct therapeutic targets. Future studies should prioritize longitudinal assessment, standardized outcome surveillance, external validation of incremental predictive value, and evaluation of treatment response before these measures can be incorporated into AF risk stratification or post-ablation management.

## 1 Introduction

Atrial fibrillation (AF) is the most common sustained cardiac arrhythmia and is closely associated with atrial structural remodeling, inflammation, oxidative stress, metabolic disturbances, and a broad range of systemic comorbidities (1, 2). In recent years, growing attention has been directed toward the role of lipid metabolism in AF. Circulating lipid profiles are well-established markers of atherosclerotic cardiovascular disease (ASCVD) risk, but they may also provide information on lipoprotein function, inflammatory burden, nutritional status, and metabolic homeostasis, all of which may be relevant to the development of an AF-susceptible atrial substrate (3, 4). Accordingly, the clinical meaning of lipid-related markers in AF may extend beyond conventional vascular risk and involve more complex links with atrial remodeling, metabolic-inflammatory status, and overall health.

Unlike their generally established relationships with atherosclerotic disease, however, circulating lipids do not show a simple or consistent linear association with AF. Several cohort studies and meta-analyses have reported that lower levels of total cholesterol (TC) and low-density lipoprotein cholesterol (LDL-C) are paradoxically associated with a higher risk of AF, a phenomenon commonly referred to as the “cholesterol paradox” (3–6). Inverse associations have also been reported for RC in several prospective and retrospective cohorts (7–9). Findings for triglycerides (TG), high-density lipoprotein cholesterol (HDL-C), apolipoproteins, lipoprotein(a) [Lp(a)], and lipid-related composite indices have likewise varied substantially across studies. This heterogeneity suggests that lipid markers should not be interpreted as uniformly harmful or protective in AF, but rather in the context of age, sex, medication use, inflammatory status, nutritional condition, metabolic burden, and coexisting diseases.

As catheter ablation has become increasingly integrated into rhythm-control strategies for AF, interest in lipid-related states has extended from disease onset to post-ablation recurrence. Recurrence after ablation is not simply a continuation of the risk processes underlying incident AF, nor can it be attributed solely to pulmonary vein reconnection, lesion discontinuity, or insufficient lesion durability. Rather, it is a treatment-response outcome shaped by the pre-existing atrial substrate, the metabolic-inflammatory environment, the tissue response to ablation, postprocedural inflammation and repair, and residual arrhythmogenic triggers (10–12). In this setting, circulating lipids and their derived indices may be clinically relevant not because they independently determine recurrence, but because they may identify patients with an adverse metabolic substrate, heightened inflammatory burden, or an unfavorable peri-ablation state.

Against this background, this review examines the available evidence on circulating lipids and lipid-related markers across two clinically distinct stages: incident AF and recurrence after catheter ablation. In contrast to previous reviews that have primarily focused on conventional lipid measures in relation to incident AF or on individual lipid-related metabolic indices, the present review does not seek to propose a single unifying pathogenic pathway. Instead, it compares the clinical meaning of lipid-related signals across long-term AF susceptibility and post-ablation rhythm outcomes. Single lipid measurements, longitudinal lipid exposure, composite indices of insulin resistance, and the background use of lipid-lowering and metabolic therapies are considered within the same stage-specific framework. This approach also enables a clearer distinction among observational associations, causal evidence, and predictive utility, while defining the clinical applications that are not yet supported by current evidence.

For this narrative review, PubMed and Web of Science were primarily searched for relevant studies published through June 2026. Search terms included “atrial fibrillation,” “lipid,” “triglyceride,” “LDL-C,” “HDL-C,” “remnant cholesterol,” “apolipoprotein,” “lipoprotein(a),” “triglyceride-glucose index,” “insulin resistance,” “catheter ablation,” and “recurrence,” used in combinations tailored to the respective topics. In addition, one Chinese master's thesis retrieved through the China National Knowledge Infrastructure database and directly evaluating the association between the apolipoprotein B/apolipoprotein A-I ratio and recurrence after radiofrequency catheter ablation was considered as supplementary evidence. The review drew on systematic reviews and meta-analyses, prospective and retrospective observational studies, Mendelian randomization analyses, mechanistic investigations, and relevant interventional studies. For post-ablation recurrence, Supplementary Table S1 summarizes original clinical observational studies directly evaluating the associations of lipid or lipid-related markers with recurrence outcomes, predominantly prospective and retrospective cohort studies. Studies unrelated to AF outcomes, studies without a clearly defined lipid or lipid-related measure, conference abstracts, and case reports were excluded. Because this was a narrative review, no formal systematic-review selection process or quantitative risk-of-bias assessment was performed.

## 2 Circulating lipid profiles and the risk of incident AF

### 2.1 TG and incident AF: From a single lipid measure to metabolic burden

The association between TG and incident AF remains uncertain, with findings varying across study designs and populations. The cross-sectional LIPIDOGRAM2015 study did not identify a consistent association between TG and AF and suggested that lipid–AF relationships may differ from conventional patterns of atherosclerotic risk (13). However, the absence of temporal information and the potential influence of age, frailty, nutritional status, medication use, and comorbidity burden limit the interpretation of cross-sectional findings.

Prospective evidence has generally suggested a positive association. The MESA and Framingham studies reported that higher baseline TG levels were associated with an increased risk of incident AF (14), whereas the KIHD study did not identify a significant association between TG and incident AF (15). In the large Swedish AMORIS cohort, higher TG levels measured in midlife remained associated with AF over follow-up extending to 30 years (16). Rather than representing only a transient lipid abnormality, TG may therefore partly capture the cumulative metabolic burden associated with long-term disturbances in glucose and lipid homeostasis.

This association should not be interpreted as evidence that elevated circulating TG directly causes AF. Higher TG commonly accompanies obesity, insulin resistance, and metabolic syndrome and may primarily indicate a broader state of systemic metabolic dysfunction. Inflammation, oxidative stress, and atrial fibrosis associated with this metabolic background may contribute to the formation of an AF-susceptible substrate. Epicardial adipose tissue (EAT), in particular, may provide an anatomical and biological interface between systemic metabolic abnormalities and local atrial remodeling (17). TG should therefore be interpreted in conjunction with adiposity, metabolic inflammation, and the local atrial adipose environment.

Disturbances in lipid handling within atrial tissue offer additional mechanistic clues. In mice, adipose triglyceride lipase (ATGL) deficiency increased susceptibility to angiotensin II–induced AF. This phenotype was accompanied by reduced peroxisome proliferator-activated receptor-α (PPAR*α*) activity, impaired fatty acid oxidation and energy metabolism, and greater inflammation, oxidative stress, and atrial fibrosis (18). Although this experimental model does not establish a direct link between circulating TG and AF in humans, it supports the possibility that impaired myocardial lipid metabolism contributes to atrial structural and electrical remodeling.

The relevance of TG to AF may thus extend beyond its conventional role in vascular risk assessment. Its circulating concentration may reflect several overlapping processes, including insulin resistance, obesity-related metabolic burden, EAT-associated inflammation, and altered myocardial lipid handling. Further studies should distinguish systemic TG levels from atrial lipid accumulation and local adipose inflammation and determine whether TG provides risk information beyond the broader metabolic context in which it is measured.

### 2.2 TC and LDL-C: the cholesterol paradox and nonlinear risk patterns

Among the lipid-related findings in AF, the inverse associations reported for TC and LDL-C are particularly difficult to reconcile with conventional ASCVD risk models. Although elevated TC and LDL-C are well-established risk factors for ASCVD, multiple observational studies and meta-analyses have linked lower baseline concentrations of these markers to a higher risk of incident AF, giving rise to the so-called “cholesterol paradox” (3, 4). These findings suggest that the relationships of TC and LDL-C with AF cannot be adequately described by a simple linear model in which higher concentrations invariably indicate greater risk.

Age, frailty, and underlying health status may partly shape this paradoxical pattern. In the Kuakini Honolulu Heart Program and Honolulu-Asia Aging Study, the apparent inverse association between TC and AF or atrial flutter was largely confined to individuals aged ≥ 75 years (19). The LIPIDOGRAM2015 study likewise suggested that age modified the association between lipid levels and AF (13). More recent work integrating grip strength, lipid measures, and genetic susceptibility further indicated that frailty may alter the risk information conveyed by conventional lipid markers (20). Accordingly, lower TC or LDL-C in older or frail individuals may not simply reflect reduced cholesterol exposure, but may also capture differences in nutritional status, multimorbidity, and overall health.

The meaning of a single cholesterol measurement may also change over time. In the Swedish AMORIS cohort, the associations of higher TC and LDL-C with lower AF risk were most evident during the earlier years of follow-up and gradually attenuated thereafter. By contrast, the associations of lower HDL-C and a higher TG/HDL-C ratio with AF were more persistent over the long term (16). This difference in temporal stability suggests that a single TC or LDL-C value may primarily characterize the circulating cholesterol phenotype at a particular stage rather than fully represent the metabolic state acting on the atria over decades. The cholesterol paradox may therefore reflect not only an unexpected direction of association, but also the limitations of static cholesterol measurements in long-term AF risk assessment.

Evidence based on repeated measurements supports this interpretation. Lee et al. reported that both lower lipid levels and greater lipid variability were associated with a higher risk of incident AF, and that long-term variability in TC and LDL-C remained associated with AF independently of baseline concentrations (5). Cholesterol-related risk signals may therefore depend on the persistence and stability of the lipid phenotype, rather than on the absolute value obtained at a single examination.

Medication exposure adds another layer of complexity. In a nationwide Korean cohort, Ahn et al. found inverse associations of TC and LDL-C with incident AF among both statin users and non-users, suggesting that statin therapy alone did not account for the cholesterol paradox (6). Nevertheless, analyses of treated populations remain susceptible to confounding by indication. Moreover, statins may influence inflammation, oxidative stress, and atrial remodeling in addition to lowering LDL-C, making it difficult to isolate the clinical meaning of the measured cholesterol concentration (6, 21).

The inverse observational pattern should not, however, be interpreted as evidence that higher cholesterol is benign or protective. Familial hypercholesterolemia (FH), which is characterized by severe and prolonged LDL-C exposure, has been associated not only with ASCVD and heart failure but also with a higher risk of AF (22). Under conditions of extreme or sustained cholesterol burden, indirect pathways involving atherosclerotic disease, valvular abnormalities, heart failure, and systemic inflammation may contribute to AF susceptibility. Thus, the relationship between TC or LDL-C and AF is more likely to be nonlinear and dependent on the duration and clinical context of exposure than uniformly inverse.

Whereas FH studies illustrate the potential consequences of long-term extreme cholesterol exposure, genetic analyses address whether TC or LDL-C itself has a direct causal effect on AF. Multivariable Mendelian randomization (MR) studies have not consistently supported an independent causal association of genetically predicted TC or LDL-C with AF after accounting for correlated lipid traits (23, 24). Drug-target MR analyses have likewise suggested that AF-related effects may differ across lipid-lowering pathways (25). These findings imply that circulating cholesterol concentrations represent only one component of a broader lipoprotein and clinical phenotype and may not fully capture the biological effects of cholesterol-related pathways on the atria.

The cholesterol paradox is therefore better understood as a context-dependent observational phenomenon than as evidence of a clearly established causal relationship. Age, frailty, nutritional status, inflammatory burden, medication use, exposure duration, and long-term lipid stability may all modify what a given TC or LDL-C concentration represents clinically. Lower TC or LDL-C associated with greater AF risk should neither be interpreted as evidence that low cholesterol directly promotes AF nor be used to infer a protective effect of higher cholesterol. These observational signals also do not undermine the need for guideline-recommended lipid-lowering therapy in patients with ASCVD or otherwise elevated cardiovascular risk.

### 2.3 HDL-C and incident AF: From cholesterol concentration to HDL function

The heterogeneous association between HDL-C and AF has increasingly shifted attention from circulating cholesterol concentration to the biological function of HDL particles. Epidemiological studies have generally associated lower HDL-C levels with a higher risk of incident AF (3, 4, 14–16). In the Swedish AMORIS cohort, lower HDL-C measured in midlife was also associated with increased long-term AF risk, and this relationship appeared more persistent over time than those observed for TC and LDL-C (16). Although these findings support a relatively consistent observational association, they do not establish that increasing HDL-C concentrations would reduce AF risk.

Nor does the available evidence support a uniformly linear protective relationship. The Japanese Suita Study reported that extremely high HDL-C levels were also associated with an increased risk of incident AF (26). This finding alone is insufficient to establish a reproducible U-shaped association, but it indicates that progressively higher HDL-C concentrations do not necessarily correspond to progressively lower AF risk. One possible explanation is that HDL-C quantifies the cholesterol carried by HDL particles without directly reflecting their number, composition, or biological activity.

Functional studies provide further insight into this distinction. Under inflammatory and oxidative conditions, the lipid and protein components of HDL may undergo oxidative modification, producing oxidized HDL (ox-HDL) with impaired antioxidant and anti-inflammatory properties. Pagonas et al. evaluated HDL oxidation by measuring HDL-associated lipid hydroperoxides and found greater HDL oxidation in patients with AF, which was associated with the presence of the arrhythmia (27). Other studies reported reduced HDL-mediated cholesterol efflux capacity in patients with AF and an association between impaired efflux and indices of left atrial remodeling (28). The ability of HDL to suppress inflammatory responses was also diminished (29). These findings suggest that AF may coexist with abnormalities in HDL oxidation, cholesterol efflux, and inflammatory regulation.

However, most evidence on HDL function has been derived from cross-sectional or case-control studies. Such designs cannot determine whether HDL dysfunction precedes AF or develops secondarily in response to the arrhythmia and its accompanying inflammatory and oxidative milieu.

Longitudinal observations suggest that HDL-related functional characteristics may also change with rhythm status. Trieb et al. reported that patients who maintained sinus rhythm after catheter ablation showed improvements in cholesterol efflux capacity, lecithin–cholesterol acyltransferase activity, apolipoprotein A-I (ApoA-I) levels, and HDL particle number compared with baseline (30). These findings indicate that at least some HDL-related measures are not fixed and may vary with cardiac rhythm or overall clinical status. Nevertheless, the follow-up sample was small, and concurrent changes in medication use, heart rate, and inflammatory status after ablation may also have contributed. The causal direction between AF and HDL dysfunction therefore remains unresolved.

HDL-C concentration alone does not adequately represent HDL particle number, composition, or function. Considered together, the findings for low HDL-C, extremely high HDL-C, and impaired HDL function raise the possibility that the heterogeneous association between HDL-C and AF partly reflects a dissociation between the cholesterol content of HDL particles and their actual biological activity. This interpretation remains hypothesis-generating because prospective studies simultaneously assessing HDL-C, HDL function, and subsequent incident AF are still lacking. Longitudinal functional measurements will be needed to determine whether HDL dysfunction forms part of an AF-susceptible phenotype or predominantly arises as a consequence of AF and its associated metabolic-inflammatory environment.

### 2.4 Remnant cholesterol and remnant lipoproteins: paradoxical signals beyond residual atherosclerotic risk

Remnant cholesterol (RC) refers to the cholesterol carried by triglyceride-rich lipoproteins (TRLs) and their remnant particles. In ASCVD, RC is generally regarded as an important component of residual risk beyond LDL-C. Its relationship with AF, however, has been far less consistent. The ARIC study associated higher RC levels with an increased risk of incident AF (31), whereas several other cohorts linked lower RC levels to a greater risk of AF (7–9). The opposing directions reported across these studies have also been highlighted in an accompanying editorial as a notable reversal in the RC–AF literature (32). The available evidence therefore points to substantial heterogeneity rather than a stable linear association in a single direction.

Differences in study populations and RC assessment may partly explain these conflicting findings. Across cohorts, variation in age, metabolic status, comorbidity burden, medication use, and baseline lipid profiles may alter the clinical meaning of a given RC value. In addition, most epidemiological studies estimate RC indirectly from TC, LDL-C, and HDL-C rather than measuring remnant particles directly. Calculated RC may therefore be influenced by TG levels, fasting status, and measurement error. It may capture not only remnant lipoprotein cholesterol burden but also broader differences in TG metabolism and the clinical characteristics of the population studied. This interpretation remains tentative and requires confirmation in studies using direct remnant lipoprotein measurements.

Current mechanistic evidence is likewise insufficient to account for the opposing directions of association. In the context of atherosclerotic disease, remnant lipoproteins have been linked to macrophage uptake, inflammatory activation, and endothelial injury (31). However, direct evidence that RC acts through these pathways within atrial tissue remains lacking. Because these processes overlap with pathways involved in AF-related remodeling, they provide indirect biological plausibility for the adverse associations observed with higher RC. The inverse associations reported for lower RC are even less well understood. Cholesterol contributes to cell membrane integrity and the regulation of membrane proteins, raising the possibility that disturbed cholesterol homeostasis could influence cellular electrophysiology. Nevertheless, this hypothesis has not been specifically tested in relation to RC, atrial tissue, or atrial ion-channel function.

Genetic studies have linked higher genetically predicted RC levels to ischemic heart disease and several other cardiovascular outcomes, although the associations vary according to the outcome, ancestry, and analytical model (33–35). Evidence specifically addressing AF remains limited, however, and does not yet establish a direct causal relationship between RC and incident AF. The pathogenic role of RC in atherosclerotic disease therefore cannot be directly extrapolated to AF. Likewise, inverse observational associations should not be interpreted as evidence that lowering RC increases AF risk.

RC should currently be viewed as a context-dependent lipid signal rather than a consistently harmful or protective factor in AF. The same calculated RC concentration may represent different combinations of remnant particle burden, TG metabolism, and underlying clinical status across populations. Future studies should incorporate direct measurements of remnant lipoproteins, repeated or longitudinal RC assessments, and AF-specific genetic and functional evidence to clarify whether the observed discrepancies mainly reflect methodological and population differences or genuinely distinct biological relationships with AF.

### 2.5 Apolipoproteins and incident AF risk: From cholesterol content to particle phenotypes

Whereas conventional lipid measures primarily quantify circulating cholesterol or TG concentrations, apolipoproteins provide complementary information on lipoprotein particle number and composition. Apolipoprotein B (ApoB) is present in very-low-density lipoproteins, remnant lipoproteins, and LDL particles, and its concentration can approximate the total number of atherogenic lipoprotein particles. ApoA-I, by contrast, is the principal structural protein of HDL and plays an important role in HDL formation, metabolism, and reverse cholesterol transport. Apolipoprotein-based assessment therefore extends lipid phenotyping in AF beyond cholesterol content toward particle burden and protein composition, although apolipoprotein concentrations alone still do not fully represent lipoprotein function.

The evidence for ApoA-I has been relatively consistent. Case-control studies have reported lower ApoA-I levels in patients with AF than in individuals without AF (36, 37), and prospective data have linked lower ApoA-I to a higher long-term risk of incident AF (15). Among patients with paroxysmal AF, reduced ApoA-I has also been observed alongside higher high-sensitivity C-reactive protein levels (37). These findings suggest that low ApoA-I may accompany an adverse lipoprotein profile and a heightened systemic inflammatory state. However, because the available evidence is derived largely from case-control studies and a single male cohort, it does not establish that reduced ApoA-I directly contributes to AF. Nor can ApoA-I concentration alone be used to infer HDL-mediated cholesterol efflux or anti-inflammatory and antioxidant function.

Associations involving ApoB and the ApoB/ApoA-I ratio are less consistent. Some case-control studies have reported lower ApoB concentrations or lower ApoB/ApoA-I ratios in patients with AF (38, 39). Prospective evidence has also suggested potential sex differences: higher ApoB was associated with a lower risk of AF mainly among women, whereas no stable association was observed among men (40). MR studies have likewise not supported a consistent direct causal effect of ApoB on AF (23, 24). Thus, the particle burden represented by ApoB may not carry the same clinical meaning in AF as it does in ASCVD, and the observed associations may be modified by sex, age, comorbidity burden, nutritional status, and correlated lipid traits.

Beyond circulating apolipoprotein concentrations, oxidative modification and the accompanying immune response may offer additional information on particle phenotype. Higher levels of immunoglobulin M (IgM) autoantibodies against the p210 oxidized epitope derived from apolipoprotein B100 (ApoB100) were associated with a lower risk of incident AF among women (41). This finding raises the possibility that humoral immune responses to oxidized lipoproteins are related to AF susceptibility. Nevertheless, the evidence comes from a specific population and a single immune marker and is insufficient to establish a direct role in AF development or to explain the sex-specific findings reported for ApoB concentrations.

Apolipoprotein studies broaden the assessment of AF-related lipid phenotypes from circulating cholesterol and TG concentrations to particle number, protein composition, and oxidative modification. The observational association between lower ApoA-I and AF appears more consistent than those involving ApoB or the ApoB/ApoA-I ratio, which remain strongly dependent on population and clinical context. This contrast further illustrates why lipid-related signals in AF cannot be interpreted solely according to particle burden or cholesterol content. Future prospective studies should assess apolipoprotein concentrations alongside particle number, oxidative modification, and lipoprotein function to determine whether these phenotypes precede AF or primarily reflect the metabolic-inflammatory environment accompanying the arrhythmia.

### 2.6 Lp(a) and AF: incident risk, population heterogeneity, and thromboembolic relevance

Lp(a) is a structurally distinct LDL-like particle in which ApoB100 is covalently linked to apolipoprotein(a) [Apo(a)] by a disulfide bond. Unlike most conventional lipid measures, circulating Lp(a) concentrations are largely genetically determined, remain relatively stable throughout adulthood, and are only modestly affected by lifestyle factors or most commonly used lipid-lowering therapies (42–44). Its biological heterogeneity extends beyond concentration alone, because Apo(a) isoform size and particle structure vary substantially among individuals. Lp(a) therefore represents a lipoprotein phenotype that differs from conventional measures of cholesterol content, yet its relationship with incident AF remains uncertain.

Some large observational cohorts and real-world analyses have associated higher Lp(a) levels with an increased risk of AF (42, 45, 46). Other prospective studies, however, have produced conflicting findings. Some found no significant association, others reported associations in the opposite direction, and still others observed only modest risk increases that did not consistently reach statistical significance (47–49). Studies of hospitalized Chinese populations have linked lower Lp(a) concentrations to AF, with a more pronounced association among women (50). Current evidence therefore supports neither higher Lp(a) as a consistent risk factor for incident AF across populations nor the interpretation that lower Lp(a) itself increases AF risk.

Differences in particle characteristics and measurement methods may partly account for this inconsistency. Lp(a) can be reported as mass concentration or particle concentration, but these measures are not interchangeable. Because Apo(a) isoform size affects the molecular mass of individual particles, similar mass concentrations may correspond to different particle numbers. Some assays are also influenced by Apo(a) isoform size, further limiting direct comparison across studies (43, 47–51). In addition, Lp(a) concentrations and Apo(a) isoform distributions vary substantially across ancestral and genetic backgrounds. The sex-specific signals observed in some cohorts likewise suggest that host characteristics may modify the association with AF. These factors plausibly contribute to between-study heterogeneity, although it remains unclear whether the conflicting results arise primarily from measurement and population differences or from genuinely distinct AF-related effects of different Lp(a) particle phenotypes.

Genetic evidence has not fully resolved these discrepancies. One study combining observational and genetic analyses reported a modest positive association between genetically predicted Lp(a) and AF (42). However, a meta-analysis did not identify a significant pooled association between genetically elevated Lp(a) and AF (51). The available evidence is therefore insufficient to establish a direct causal effect of Lp(a) on incident AF. Its established pathogenic role in atherosclerotic disease should not be automatically extrapolated to AF development.

The absence of a stable directional association highlights the particular complexity of Lp(a) in AF. Even for a strongly heritable lipoprotein marker, a single circulating concentration may not adequately characterize the particle phenotype relevant to AF. Future studies should first define the exposure more precisely by distinguishing mass from particle concentration, incorporating Apo(a) isoform information, and comparing associations across sexes and genetic backgrounds. Such approaches may help determine whether the present inconsistencies mainly reflect methodological and population heterogeneity or whether distinct Lp(a) particle phenotypes have genuinely different biological relationships with AF.

Beyond the risk of AF onset, Lp(a) may also have clinical relevance to thromboembolic risk in patients with established AF. Kamili et al. reported an association between higher Lp(a) levels and left atrial appendage thrombus or spontaneous echo contrast in patients with nonvalvular AF (52). More recent work has suggested that combining Lp(a) with left atrial appendage morphology and clinical variables may improve thromboembolic risk stratification (53). These findings, however, primarily concern thrombogenicity after AF has developed and should not be interpreted as evidence that Lp(a) contributes to the initial onset of the arrhythmia.

**Figure 1:**
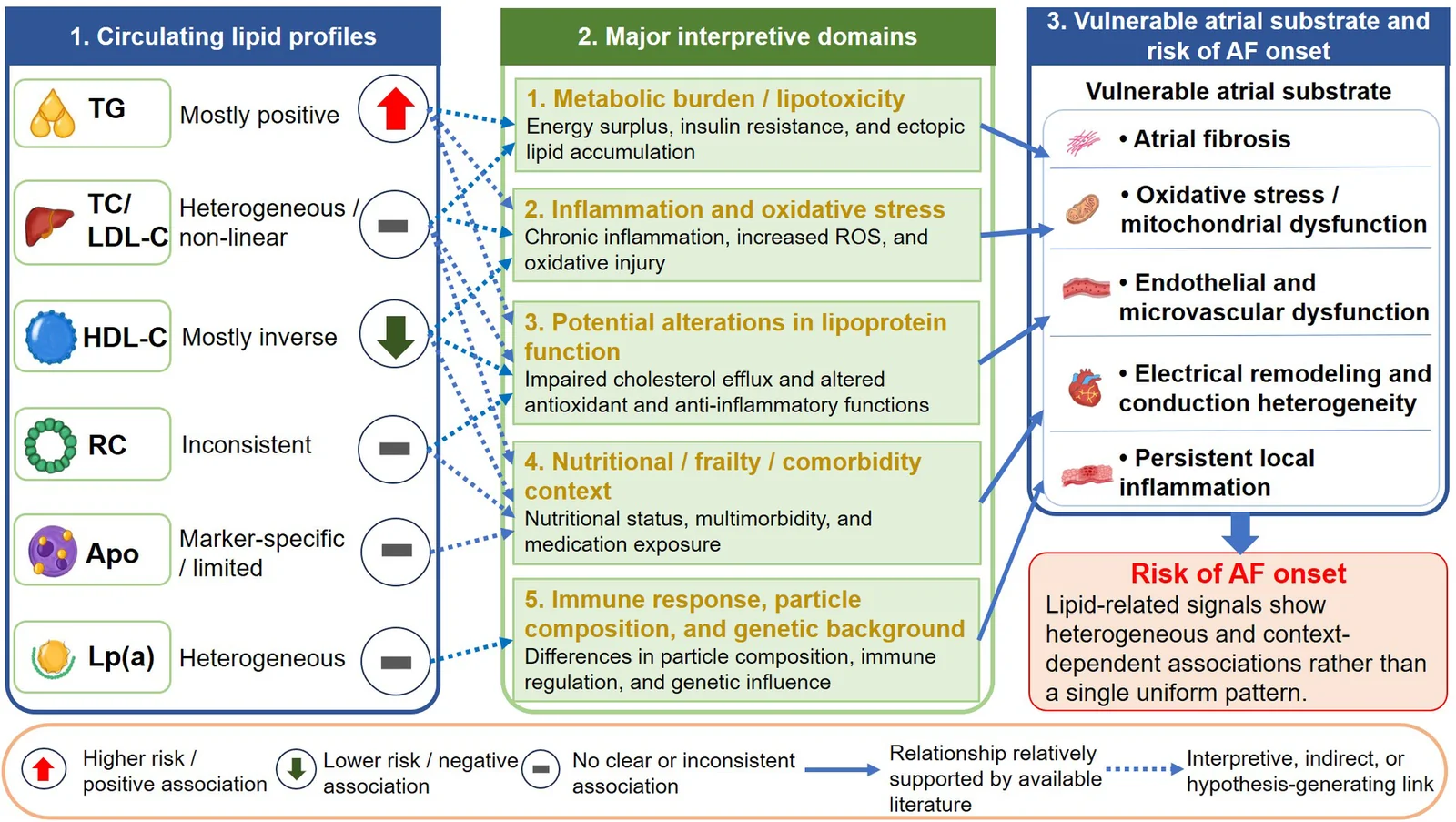
From circulating lipid profiles to vulnerable atrial substrate and risk of AF onset. Solid arrows indicate relationships relatively supported by the available literature, whereas dashed arrows indicate indirect, interpretive, or hypothesis-generating links that have not been directly established.

Taken together, the evidence reviewed above indicates that circulating lipid-related signals show heterogeneous and context-dependent associations with incident AF and may reflect multiple aspects of metabolic-inflammatory status and the atrial substrate. These relationships are summarized schematically in Figure 1.

## 3 From incident risk to ablation outcomes: the peri-ablation relevance of lipid-related states

The evidence reviewed above indicates that circulating lipids do not show a stable, unidirectional association with incident AF. The direction and strength of these associations vary across lipid markers and study populations, suggesting that their clinical meaning is shaped by the broader metabolic and clinical context (3–6). Circulating lipid profiles may therefore be more appropriately interpreted as integrated signals of metabolic-inflammatory status than as isolated causal risk factors.

This perspective provides a different framework for considering recurrence after catheter ablation. Studies of incident AF primarily address an individual's long-term susceptibility to developing the arrhythmia in the setting of cumulative metabolic exposure, comorbidity burden, and progressive atrial remodeling. Post-ablation recurrence, by contrast, occurs in patients with established AF and varying degrees of atrial substrate abnormality after a defined rhythm-control intervention and procedure-related tissue injury. It is therefore more appropriately regarded as an outcome of post-treatment rhythm maintenance (10–12, 54). Accordingly, the direction of lipid associations observed in incident AF cannot be directly extrapolated to post-ablation recurrence, and lipid-related markers in this setting should not be assumed to exert a direct effect on recurrence.

From a peri-ablation perspective, the association between lipid-related states and recurrence may be understood across three interconnected phases: the pre-ablation substrate, lesion formation, and postprocedural repair. Before ablation, obesity, insulin resistance, chronic low-grade inflammation, and abnormalities of EAT may collectively reflect an adverse metabolic-inflammatory atrial environment. In patients undergoing thoracoscopic ablation for advanced AF, atrial EAT inflammatory characteristics differed according to AF type, whereas recurrence was associated with larger adipocytes rather than higher inflammatory cell counts (55). In a separate RFCA cohort, greater left atrial EAT and a larger low-voltage area burden were independently associated with post-ablation recurrence (56). These findings do not establish that circulating lipids directly produce atrial substrate abnormalities, but they provide indirect support for why lipid-related markers may be associated with recurrence.

During ablation, acute electrical isolation does not necessarily indicate that durable lesions have been established. Analyses related to the DECAAF II trial showed that lesions created by radiofrequency energy were not uniformly converted into stable scar, and that baseline atrial fibrosis may influence lesion coverage and subsequent recurrence (11). Ablation efficacy may therefore depend not only on energy delivery but also on the pre-existing condition of atrial tissue and the subsequent process of scar formation. This does not imply that lipid abnormalities directly impair lesion formation; rather, lipid-related metabolic states may coexist with complex atrial characteristics such as fibrosis, low-voltage areas, and abnormal epicardial adipose tissue.

After ablation, dynamic processes including tissue edema, inflammation, oxidative stress, autonomic changes, and injury repair may contribute to early recurrence. Over longer follow-up, pulmonary vein reconnection, incomplete scar maturation, and persistent structural or electrophysiological remodeling may become more relevant to late recurrence (10, 12, 54). Post-ablation recurrence is therefore better understood as a treatment-related outcome jointly influenced by the pre-existing atrial substrate, lesion durability, and the trajectory of postprocedural repair, rather than as the direct consequence of a single lipid measure or ablation technique.

Within this framework, the potential value of circulating lipids and related indices in ablation populations may lie less in independently determining recurrence risk than in indicating preprocedural metabolic burden, inflammatory background, and a potentially recurrence-prone atrial state. The following section reviews the clinical evidence linking lipid-related markers to recurrence after catheter ablation, with attention to the study population, ablation modality, recurrence definition, and follow-up strategy used in each investigation.

## 4 Circulating lipids and recurrence after AF catheter ablation

### 4.1 Scope of evidence, ablation modalities, and definitions of recurrence

This section considers AF catheter ablation as the overall clinical setting. Most of the available evidence is derived from cohorts undergoing radiofrequency catheter ablation (RFCA), with additional findings from studies of cryoballoon ablation and pulsed field ablation (PFA). Because these modalities differ in their mechanisms of energy delivery, lesion formation, tissue injury, and potentially their peri-procedural inflammatory responses, the specific ablation technique used in each study is identified where relevant. Findings obtained with one modality should not be assumed to apply directly to patients treated with another.

Definitions and methods of detecting recurrence also vary across studies. For the purposes of this review, early recurrence refers to atrial arrhythmias occurring within the blanking period specified by the original study, whereas late recurrence refers to events occurring after that period. Definitions of very late or long-term recurrence are retained as reported in the respective studies. Although most included studies used a 3-month blanking period, they differed in the duration of the blanking period, definitions of early and late recurrence, minimum arrhythmia-duration thresholds, types of atrial arrhythmias included, and length of follow-up. Accordingly, the original outcome definitions were preserved, and recurrence endpoints were not compared directly without consideration of these methodological differences.

Post-ablation recurrence was identified using various approaches, including scheduled 12-lead electrocardiograms, ambulatory electrocardiographic monitoring, symptom-triggered examinations, outpatient follow-up, and continuous or implantable rhythm monitoring. The frequency and duration of monitoring directly influence the detection of asymptomatic recurrence. Clinical recurrence detected by intermittent electrocardiographic assessment is therefore not fully equivalent to AF episodes or AF burden documented by continuous monitoring.

The design, patient characteristics, ablation modality, recurrence definition and blanking period, rhythm-monitoring strategy, follow-up duration, lipid-related marker, key adjustment variables, and principal findings of the post-ablation studies are summarized in Supplementary Table S1 to facilitate assessment of their comparability.

### 4.2 TG and TyG-related indices: insulin resistance and metabolic burden

Evidence linking a single TG measurement to recurrence after catheter ablation remains inconsistent. An early prospective study found that TG was not independently associated with recurrence, whereas inverse associations of TC and LDL-C were observed among women (57). By contrast, a retrospective RFCA cohort reported higher recurrence risks in both the intermediate- and high-TG groups compared with the low preprocedural TG group (58). These divergent findings may reflect differences in study design, patient characteristics, baseline glucose and lipid metabolism, and covariate adjustment. In patients undergoing ablation, TG alone may capture only one component of the metabolic background and may be insufficient to characterize the broader metabolic state associated with recurrence.

The triglyceride–glucose index (TyG), which integrates TG and fasting plasma glucose (FPG), is commonly used as a surrogate marker of insulin resistance (IR). Associations between TyG and post-ablation recurrence have been relatively consistent across the available studies. Among patients without diabetes undergoing first-time RFCA, a higher preprocedural TyG was associated with late recurrence (59). In another cohort of patients undergoing first-time RFCA, TyG was also independently associated with early recurrence within the blanking period (60). Compared with TG alone, the combined assessment of lipid and glycemic information may therefore identify an adverse pre-ablation metabolic state more readily. Nevertheless, the statistical association between TyG and recurrence does not establish that IR directly affects lesion formation, tissue repair, or the recurrence process.

Subsequent studies have incorporated adiposity and broader metabolic burden into TyG-based assessment. The TyG–body mass index (TyG-BMI) combines TyG with body mass index (BMI), whereas the metabolic score for insulin resistance (METS-IR) integrates multiple metabolic and anthropometric variables. In a two-center retrospective study, TyG, TyG-BMI, and METS-IR were independently associated with recurrence after RFCA, with TyG-BMI showing the highest, although still modest, discrimination among the four insulin-resistance indices examined (61). In a mediation analysis of 910 patients undergoing RFCA, left atrial diameter (LAD) accounted for part of the association between TyG-BMI and recurrence (62). This result provides statistical support for a link between metabolic burden and left atrial remodeling, but it does not demonstrate that metabolic abnormalities cause recurrence through left atrial enlargement.

Most of these indices are based on a single preprocedural or peri-procedural measurement and therefore provide limited information on the persistence of metabolic abnormalities before and after ablation. More recent studies have consequently examined cumulative TyG exposure and longitudinal TyG trajectories. In RFCA cohorts, persistently high TyG trajectories and greater cumulative TyG exposure during the first 3 months after ablation were associated with a higher risk of recurrence (63, 64). These approaches incorporate a temporal dimension that is absent from a single baseline value. Whether they improve risk assessment beyond baseline TyG and established recurrence-related factors, however, requires direct comparison and external validation.

A recent systematic review and meta-analysis further reported that surrogate indices of IR, including TyG, the homeostasis model assessment of insulin resistance (HOMA-IR), and METS-IR, were generally associated with a higher risk of AF recurrence after catheter ablation, whereas the association for the TG/HDL-C ratio was not consistent (65).

The contrast between single TG measurements and composite or longitudinal indices suggests that recurrence may be more closely related to sustained overall metabolic burden than to TG concentration at one time point. TyG, TyG-BMI, METS-IR, HOMA-IR, and dynamic TyG measures have shown comparatively more consistent signals, with partial support from pooled evidence. Their current value, however, lies primarily in identifying patients with an adverse metabolic background rather than demonstrating that lowering TG or TyG will reduce recurrence. Future studies should determine whether peri-ablation metabolic trajectories provide information beyond AF type, left atrial structure, and comorbidity burden, and whether improvement in metabolic status is accompanied by a corresponding reduction in recurrence. Such evidence will be needed to distinguish peripheral risk markers from potentially modifiable treatment-response phenotypes.

### 4.3 TC and LDL-C: the cholesterol paradox and nonlinear associations with recurrence

The relationship of TC and LDL-C with recurrence after catheter ablation remains unsettled (57, 66–68). Sex-specific findings and variation across different concentration ranges may help explain some of the apparently conflicting results.

Sex stratification is one of the more notable features of the available evidence. In a prospective study of 287 patients, lower TC and LDL-C levels were associated with a higher risk of post-ablation recurrence, but these associations were primarily observed among women; neither HDL-C nor TG showed an independent association (57). A single-center study of 412 patients undergoing RFCA reported a similar pattern. Associations of TC and LDL-C with recurrence were not consistent in the overall cohort, whereas associations were observed among women (67). These findings suggest that low TC or LDL-C is unlikely to represent a universal risk marker across all ablation populations and that its clinical relevance may differ by sex. The biological basis of this apparent interaction, however, remains unclear.

The association between LDL-C and recurrence may also vary across concentration ranges. A dose–response analysis from a prospective RFCA cohort identified an L-shaped relationship, with an inflection point at approximately 3.20 mmol/L (66). Below this level, lower LDL-C was associated with a higher risk of recurrence, whereas the association became attenuated above the inflection point. This finding suggests that a conventional linear analysis may obscure an inverse association confined to the lower LDL-C range. Nevertheless, the proposed inflection point was derived from a single study and should not be regarded as a generally applicable clinical threshold. Nor does the finding imply that increasing LDL-C would reduce post-ablation recurrence.

The direction of association has not been consistent across studies. Another investigation reported that higher LDL-C levels were associated with an increased risk of recurrence after RFCA (68). Differences in patient selection, AF type and duration, the extent of left atrial remodeling, lipid-lowering treatment, and baseline lipid distributions may have contributed to these divergent results. At present, the evidence is therefore more consistent with substantial population heterogeneity than with a uniform risk direction.

The available data do not support a stable linear relationship between TC or LDL-C and post-ablation recurrence. The same lipid concentration may carry different implications according to sex, the range of exposure, and the broader clinical context. In ablation populations, preprocedural TC and LDL-C may also reflect previous lipid-lowering therapy, treatment adherence, and underlying cardiovascular disease burden. Future studies should therefore validate the reported female-specific and L-shaped associations in independent cohorts and incorporate repeated lipid measurements together with detailed treatment information. Distinguishing persistently low concentrations from treatment-related reductions and changes occurring during follow-up will be important for determining whether TC and LDL-C provide independent risk-stratification information or merely reflect treatment exposure and underlying disease severity.

### 4.4 HDL-C–related composite indices: interpretive value in the context of inflammation and oxidative stress

Direct assessments of conventional lipid measures have not identified HDL-C as a stable independent marker of recurrence after catheter ablation (57, 67). By contrast, composite ratios incorporating HDL-C have repeatedly shown associations with recurrence. The most extensively studied of these is the monocyte-to-HDL-C ratio (MHR).

Canpolat et al. first reported that a higher preprocedural MHR was associated with an increased risk of AF recurrence after cryoballoon ablation (69). Chen et al. subsequently observed a similar association with late recurrence among patients with paroxysmal AF undergoing RFCA (70). The recurrence signals reported across different ablation modalities and patient populations suggest that MHR may convey information not captured by HDL-C alone. Its potential value lies not simply in replacing HDL-C with a ratio, but in combining a marker of monocyte-related inflammation with a lipid measure to characterize a broader inflammatory–lipid phenotype.

MHR should not, however, be interpreted as a direct measure of the biological balance between inflammation and HDL function. An elevated value may result from a higher monocyte count, a lower HDL-C concentration, or changes in both components, and these patterns may not have equivalent biological implications. MHR is therefore better regarded as a composite inflammatory–lipid marker than as evidence that increased monocytes or reduced HDL-C directly cause post-ablation recurrence.

Several studies have further examined whether MHR adds information to established structural or clinical variables. In an RFCA cohort of patients with paroxysmal AF, the combination of MHR and LAD showed better discrimination for recurrence than either measure alone (71). In a retrospective cohort including patients treated with RFCA or cryoballoon ablation, adding MHR to the APPLE score was also associated with improved model performance for late recurrence (72). These findings suggest that MHR may provide information complementary to left atrial structure and conventional clinical variables. Nevertheless, improvements observed within the original cohorts require external validation before their generalizability can be established, and they should not be regarded as mechanistic evidence linking HDL-C to atrial remodeling.

The uric acid-to-HDL-C ratio (UHR) extends the same composite-marker approach. Dervis et al. found that a higher UHR was associated with arrhythmia recurrence in a cohort that included both radiofrequency and cryoballoon ablation (73). In a larger cryoballoon ablation cohort, Yang et al. further reported associations of elevated UHR with both late and very late recurrence (74). As with MHR, the potential relevance of UHR lies in integrating two routinely available measures from different biological domains rather than in isolating the effect of either uric acid or HDL-C. UHR does not directly quantify oxidative stress, nor does it demonstrate that uric acid-related injury outweighs the biological functions of HDL.

Current evidence does not support HDL-C alone as a consistent marker of post-ablation recurrence, whereas MHR and UHR show some potential for risk stratification. This contrast suggests that the information carried by HDL-C in ablation populations may become more apparent when considered alongside other biological dimensions. It does not, however, establish that these ratios represent specific pathological mechanisms. Future studies should compare the independent contributions of MHR, UHR, and their individual components; determine whether they improve discrimination beyond AF type, left atrial structure, and established clinical models; and evaluate whether peri-ablation changes track with recurrence. Integrating these measures with assessments of HDL particle number, cholesterol efflux capacity, and anti-inflammatory or antioxidant function may help distinguish convenient clinical markers from indices that more closely reflect functional states relevant to ablation outcomes.

### 4.5 Other lipoprotein-related markers: exploratory signals at the particle level

Beyond conventional lipid measures and HDL-C–related composite indices, RC, apolipoproteins, and Lp(a) have also been investigated in relation to rhythm outcomes after AF ablation. The available evidence remains limited and does not support firm conclusions. Nevertheless, these studies extend the focus from routine lipid concentrations to remnant lipoprotein metabolism, apolipoprotein profiles, and genetically influenced lipoprotein phenotypes.

For remnant lipoproteins, a single-center retrospective study of 320 patients undergoing first-time RFCA reported 103 recurrences during 1 year of follow-up. Patients with recurrence had lower remnant-like particle cholesterol (RLP-C) levels than those who remained free of recurrence, and multivariable Cox analysis showed that each 0.1 mmol/L increase in RLP-C was associated with a lower recurrence risk (75). This direction differs from the adverse association generally observed for higher remnant cholesterol in ASCVD and resembles the inverse signals reported for low TC and LDL-C in some ablation cohorts. However, the finding is derived from a single study, and the extent to which patient selection, lipid-lowering treatment, or baseline lipid distributions influenced the association remains unclear. Moreover, RLP-C reflects the cholesterol content of remnant-like particles rather than their number or metabolic activity. The result should therefore be regarded as a paradoxical association requiring independent replication, rather than evidence that higher RLP-C protects against recurrence.

Apolipoprotein studies have followed two distinct lines of investigation: short-term peri-procedural changes and baseline risk assessment. A small prospective study examined changes in apolipoprotein profiles after cryoballoon ablation and found alterations at 24 h in ApoA-I, apolipoprotein A-II (ApoA-II), ApoB, apolipoprotein C-I (ApoC-I), apolipoprotein D (ApoD), apolipoprotein E (ApoE), and apolipoprotein J (ApoJ). Among these markers, ApoJ showed some discriminatory ability in the assessment of cryoablation effectiveness (76). These observations suggest that ablation may be accompanied by acute changes in circulating apolipoproteins, but the short observation period and absence of a long-term recurrence endpoint preclude conclusions about their relevance to subsequent rhythm outcomes.

A separate single-center study evaluated the baseline ApoB/ApoA-I ratio in 232 patients undergoing RFCA, of whom 60 experienced recurrence during follow-up. Multivariable analysis identified a higher ApoB/ApoA-I ratio and LAD as independently associated with recurrence (77). However, the area under the curve (AUC) for the ratio was only 0.611, indicating limited ability to distinguish between patients with and without recurrence when used alone. At present, it may be more appropriately considered a supplementary variable than a stand-alone predictive marker.

Evidence for Lp(a) has largely emerged from selected ablation modalities and specific patient populations. In a study of patients undergoing PFA, those with recurrence had higher Lp(a) levels, and recurrence risk increased across higher Lp(a) levels (78). Another prognostic study in patients with patent foramen ovale and paroxysmal AF also included Lp(a) among the candidate variables used to assess recurrence after ablation (79). The latter finding primarily indicates that Lp(a) has been explored within a specialized prediction model; it does not establish stable independent predictive value or incremental performance. Given the restrictions imposed by ablation modality, sample size, and patient selection, it remains uncertain whether these Lp(a)-related signals apply to the broader AF ablation population.

The evidence for RC, apolipoproteins, and Lp(a) after ablation remains exploratory and represents several distinct research questions. The inverse association reported for RLP-C requires replication; the relationship between acute peri-procedural apolipoprotein changes and long-term rhythm outcomes remains undefined; the incremental value of the ApoB/ApoA-I ratio beyond established clinical factors requires further evaluation; and the Lp(a) signal observed in selected settings must be tested in more representative ablation cohorts.

**Figure 2:**
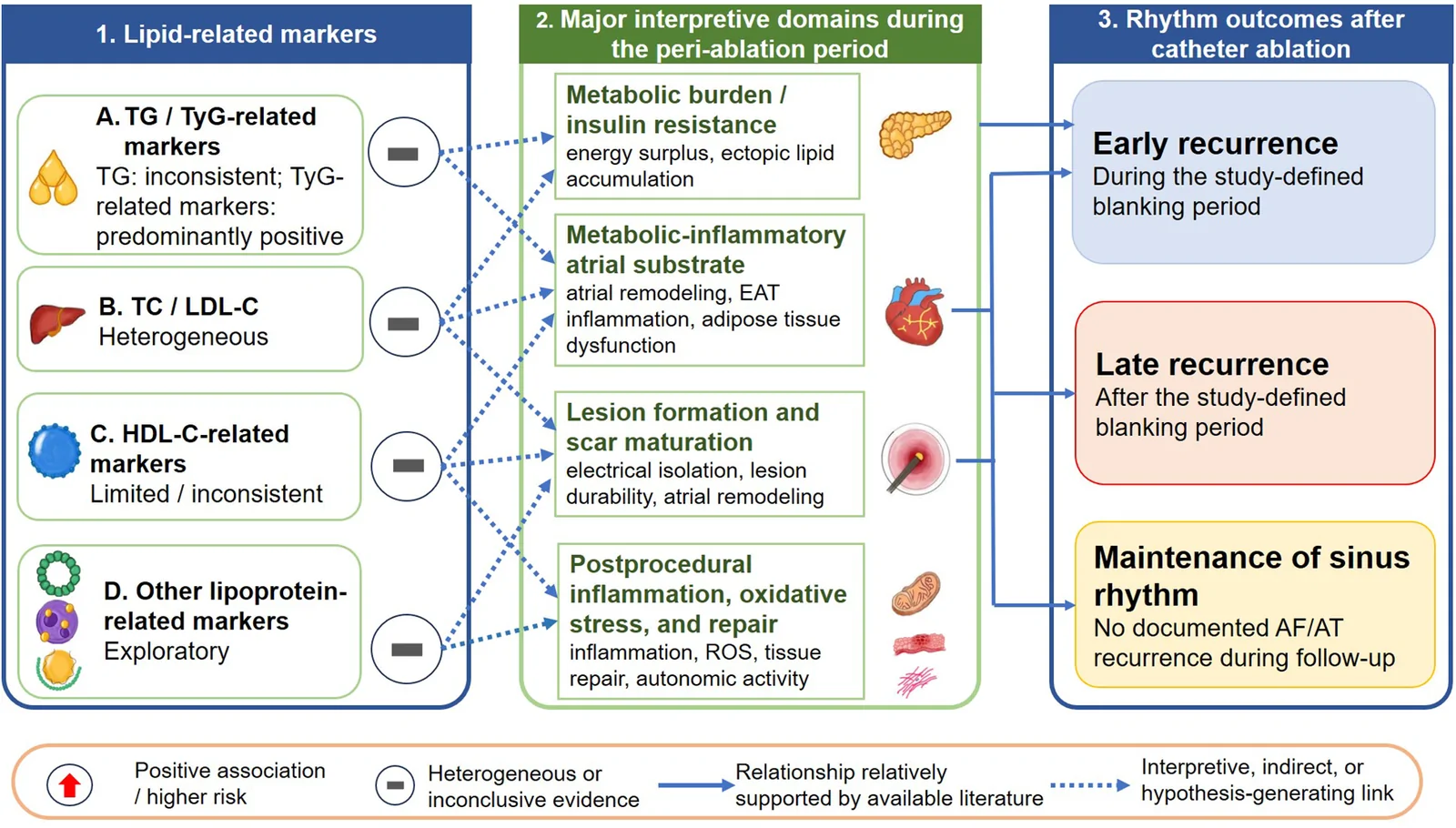
Lipid-related states during the peri-ablation period and rhythm outcomes after catheter ablation. Solid arrows indicate relationships relatively supported by the available literature, whereas dashed arrows indicate indirect, interpretive, or hypothesis-generating links that have not been directly established. Early and late recurrence were defined according to the blanking period specified in each original study. Most included studies used a 3-month blanking period, although the duration and recurrence definitions varied across studies.

Taken together, the evidence reviewed for incident AF and post-ablation recurrence highlights substantial heterogeneity in the direction and clinical meaning of lipid-related associations across different clinical stages and markers. A comparative summary of the evidence for the major lipid and lipid-related markers is provided in Table 1. Figure 2 further illustrates how these lipid-related states may be interpreted within the peri-ablation framework, from the pre-ablation atrial substrate and lesion formation to postprocedural repair and subsequent rhythm outcomes.

**Table 1:** Summary of lipid and lipid-related markers in incident AF and post-ablation recurrence.

Lipid or lipid-related marker	Evidence for incident AF	Evidence for post-ablation recurrence	Association pattern	Major limitations	Interpretation
TG and TG-related insulin resistance indices	Higher TG has been associated with a greater risk of incident AF in several prospective cohorts, although findings are not entirely consistent.	Associations for TG alone are inconsistent; TyG, TyG-BMI, METS-IR, HOMA-IR, and longitudinal TyG measures have shown comparatively more consistent positive associations with recurrence.	TG alone: inconsistent; insulin resistance–related indices: predominantly positive.	Predominantly observational evidence; heterogeneous recurrence definitions, rhythm-monitoring strategies, diabetes status, ablation modalities, and covariate adjustment.	These measures may reflect overall metabolic burden and insulin resistance more than a direct effect of TG concentration.
TC and LDL-C	Lower TC and LDL-C have been associated with a higher risk of incident AF in multiple studies, giving rise to the “cholesterol paradox.”	Findings are inconsistent: inverse associations for low TC or LDL-C, an L-shaped relationship, and positive associations for higher LDL-C have all been reported.	Heterogeneous or nonlinear.	Age, frailty, nutritional status, lipid-lowering treatment, sex, and selection bias may influence the observed associations.	These findings should not be interpreted as evidence that higher cholesterol is protective and should not alter guideline-recommended lipid-lowering therapy.
HDL-C and HDL-C–related composite indices	Lower HDL-C has generally been associated with a higher risk of incident AF; some studies also suggest that extremely high HDL-C may be associated with increased risk.	HDL-C alone is not a stable marker of recurrence, whereas MHR and UHR have shown some risk-stratification value in selected ablation cohorts.	HDL-C alone: inconsistent; MHR and UHR: predominantly positive risk signals.	MHR and UHR do not directly measure HDL function, inflammation, or oxidative stress, and external validation remains limited.	These indices may capture combined inflammatory–lipid or oxidative stress–lipid states rather than the effect of HDL-C concentration itself.
RC and RLP-C	Associations between RC and incident AF are inconsistent, with both higher- and lower-RC risk signals reported across different cohorts.	Evidence is limited; one RFCA study reported that lower RLP-C was associated with a higher risk of recurrence.	Inconsistent, with possible inverse signals.	Few studies are available; calculated RC is not equivalent to directly measured remnant lipoproteins, and populations and measurement methods differ substantially.	These findings remain exploratory and should not be interpreted directly within the ASCVD residual-risk framework.
Apolipoproteins	Lower ApoA-I has shown a relatively consistent association with prevalent or incident AF, whereas findings for ApoB and the ApoB/ApoA-I ratio are more heterogeneous.	Evidence for recurrence is scarce; the ApoB/ApoA-I ratio has shown only limited exploratory associations and modest discrimination when used alone.	ApoA-I: predominantly inverse; ApoB-related measures: uncertain.	Recurrence studies are few and generally small; circulating apolipoprotein concentrations do not directly represent lipoprotein particle function.	Apolipoprotein measures may complement assessment of lipoprotein particle phenotypes but are currently insufficient to guide clinical decisions.
Lp(a)	Evidence relating Lp(a) to incident AF is heterogeneous and may be influenced by population, sex, assay method, and Apo(a) isoform characteristics.	Evidence for recurrence is very limited and is derived mainly from exploratory studies of PFA or selected patient populations.	Uncertain; possible positive signals in selected settings.	Sample sizes are limited, study settings are highly selected, and substantial differences exist in measurement methods and population backgrounds.	Lp(a) cannot currently be regarded as a stable recurrence marker in the general AF ablation population, and evidence for incident AF, thromboembolic risk, and recurrence should not be conflated.

AF, atrial fibrillation; ASCVD, atherosclerotic cardiovascular disease; TG, triglyceride; TC, total cholesterol; LDL-C, low-density lipoprotein cholesterol; HDL-C, high-density lipoprotein cholesterol; RC, remnant cholesterol; RLP-C, remnant-like particle cholesterol; TyG, triglyceride-glucose index; TyG-BMI, triglyceride-glucose index–body mass index; METS-IR, metabolic score for insulin resistance; HOMA-IR, homeostasis model assessment of insulin resistance; MHR, monocyte-to-HDL-C ratio; UHR, uric acid-to-HDL-C ratio; ApoA-I, apolipoprotein A-I; ApoB, apolipoprotein B; ApoB/ApoA-I, apolipoprotein B/apolipoprotein A-I ratio; Lp(a), lipoprotein(a); Apo(a), apolipoprotein(a); RFCA, radiofrequency catheter ablation; PFA, pulsed field ablation.

## 5 Integrated interpretation of lipid-related associations in AF: heterogeneity, treatment context, and clinical implications

### 5.1 Sources and interpretation of heterogeneity in lipid-related associations

The evidence summarized in the preceding sections shows that circulating lipids and lipid-related indices have not demonstrated a stable or uniform direction of association with either incident AF or recurrence after catheter ablation. Associations with incident AF differ across individual lipid measures in both direction and effect size, while studies of post-ablation recurrence also show heterogeneity across markers and study populations (3, 4, 65). These findings may reflect genuine differences across clinical stages and patient backgrounds, as well as variation in study methodology, and should not be reduced to the conclusion that the available studies are simply contradictory.

Patient characteristics and clinical stage are important sources of this variation. Age, sex, obesity, diabetes, heart failure, renal function, nutritional status, and inflammatory burden may influence both circulating lipid levels and AF-related outcomes. For example, the association between cholesterol and AF has varied according to age, follow-up period, and statin-use status (6, 13, 16). Incident AF primarily reflects long-term susceptibility to developing the arrhythmia, whereas post-ablation recurrence occurs in patients with established AF and an abnormal atrial substrate and is additionally influenced by ablation-related injury, lesion formation, and postprocedural inflammation and repair (10–12, 54). Associations observed for one outcome therefore cannot be directly transferred to the other.

Differences in the assessment of lipid exposure and AF outcomes may further contribute to between-study heterogeneity. Most studies rely on a single baseline measurement, which cannot adequately capture long-term changes in lipid status. Composite indices incorporate glycemia, body size, or other metabolic information, meaning that their associations cannot be attributed solely to their lipid components. Cumulative measures and trajectory-based indices add information on the duration and direction of exposure, but their grouping strategies and statistical models still require validation across independent populations (5, 61, 63–65). Outcome ascertainment also varies. Differences in the sources used to diagnose incident AF and, among ablation studies, in blanking-period definitions, follow-up duration, ablation modality, and rhythm-monitoring intensity may affect both event detection and estimated effect sizes (54, 65).

The evidence base remains predominantly observational, with substantial variation in covariate selection, cutoff determination, and statistical modeling. Residual confounding and reverse causation therefore cannot be fully excluded. MR provides complementary genetic evidence, but lifelong genetically determined exposure is not equivalent to a single circulating lipid measurement, and different lipid-lowering targets represent distinct biological pathways. MR findings should therefore not be viewed as direct substitutes for clinical observational evidence (23–25). Similarly, systematic reviews and meta-analyses can improve the precision of pooled estimates and characterize between-study heterogeneity, but their conclusions remain constrained by the design limitations and residual confounding of the original studies. Different forms of evidence should thus be regarded as complementary rather than interchangeable.

Variation in racial or regional composition, the proportions of paroxysmal and persistent AF, and patient-selection procedures may further limit comparability across studies.

The inconsistent associations between circulating lipids and AF-related outcomes do not currently justify classifying any single marker as a uniformly harmful or protective factor. Interpretation requires consideration of the population studied, the clinical stage, the form and duration of lipid exposure, outcome ascertainment, and study design. Lipid-lowering and metabolic therapies may further alter both measured lipid levels and AF outcomes, adding another layer of complexity that is considered in the following section.

### 5.2 Influence of treatment context on the interpretation of lipid signals

Treatment exposure is an important consideration when interpreting associations of lipid and lipid-related measures with either incident AF or recurrence after catheter ablation. Patients with AF frequently have coexisting ASCVD, diabetes, obesity, heart failure, or other cardiometabolic conditions, and their lipid profiles may be influenced by lipid-lowering therapy, glucose-lowering medication, weight-loss interventions, and comprehensive risk-factor management. Consequently, measured levels of TC, LDL-C, TG, HDL-C, or TyG do not necessarily represent untreated biological exposure. They may simultaneously reflect disease severity, treatment indication, therapeutic response, adherence, and residual metabolic risk.

#### 5.2.1 Treatment context and the interpretation of incident AF evidence

Among potential treatment-related influences, lipid-lowering therapy—particularly statin use—has received the greatest attention. Observational findings remain inconsistent. Some cohorts have reported a modestly higher risk of incident AF among users of cholesterol-lowering medications, but treated individuals are often older and have a greater burden of diabetes, hypertension, and cardiovascular disease. Moreover, many studies record medication use only at baseline and lack information on treatment intensity, adherence, and changes during follow-up (21). Medication use may therefore indicate both lipid modification and a higher underlying cardiometabolic disease burden. Conversely, inverse associations of TC and LDL-C with incident AF have been observed among both statin users and non-users, indicating that the cholesterol paradox cannot be explained entirely by statin therapy (6). The available evidence neither demonstrates that statins universally prevent incident AF nor supports the conclusion that statin treatment or treatment-associated low LDL-C increases AF risk.

Evidence from other lipid-lowering pathways further illustrates that reductions in lipid concentrations do not correspond directly to changes in AF risk. Studies involving ezetimibe have largely been conducted in selected populations at high cardiovascular risk and often involve combination therapy, making its independent association with AF difficult to determine (80). Fibrates are frequently grouped with other lipid-lowering agents in observational analyses, and studies specifically designed to evaluate incident AF as an outcome are scarce. These therapies are therefore more appropriately considered components of the treatment background that require documentation and adjustment than established interventions for AF prevention.

Genetic and pharmacological evidence relating to proprotein convertase subtilisin/kexin type 9 (PCSK9) inhibition is also not fully concordant. Drug-target MR analyses have suggested that genetically proxied PCSK9 inhibition may be associated with a lower risk of AF (25). In contrast, a secondary analysis of a randomized trial in patients with acute coronary syndrome found that PCSK9 inhibition substantially reduced LDL-C but did not significantly alter the incidence of AF (81). Lifelong genetically determined target exposure is not equivalent to pharmacological treatment initiated at a particular clinical stage. These findings also suggest that marked LDL-C reduction alone does not necessarily translate into a lower risk of AF.

The evidence for omega-3 fatty acid preparations is more extensive but depends strongly on dose, formulation, and population. Eicosapentaenoic acid (EPA) and docosahexaenoic acid (DHA) may represent habitual dietary intake and circulating fatty acid status or may be administered as high-dose prescription therapies. In general primary-prevention populations, lower-dose combined EPA and DHA supplementation did not significantly affect incident AF risk (82). By contrast, randomized trials of high-dose EPA/DHA formulations or purified EPA in patients with elevated TG or high cardiovascular risk have repeatedly reported an increased AF signal (83–86). Meta-analyses of randomized trials have similarly associated omega-3 supplementation with higher AF risk, with a potentially stronger signal in higher-dose studies. However, this dose-related pattern is based largely on comparisons across trials rather than randomized dose comparisons within the same study (87).

These findings should not be summarized as evidence that omega-3 fatty acids universally increase AF risk. Circulating or tissue omega-3 biomarkers mainly reflect long-term dietary exposure and endogenous metabolism and are not equivalent to short-term treatment with high-dose pharmaceutical preparations. In cohort studies, higher levels of DHA, docosapentaenoic acid, and combined EPA plus DHA have instead been associated with a lower risk of incident AF, whereas the association for EPA alone has been less clear (88). Habitual intake, circulating biomarkers, low-dose supplementation, and high-dose prescription formulations therefore represent distinct exposures, and evidence from one setting should not be directly extrapolated to another.

Metabolic therapies that do not primarily target lipids may also influence incident AF. Sodium–glucose cotransporter 2 inhibitors (SGLT2i) and glucagon-like peptide-1 receptor agonists (GLP-1RA), for example, affect multiple aspects of cardiometabolic status. A *post hoc* analysis of a randomized trial in patients with type 2 diabetes and high cardiovascular risk associated dapagliflozin with fewer AF or atrial flutter events (89). Meta-analyses of randomized trials have also suggested that SGLT2i may reduce AF risk, although findings have not been uniform across underlying diseases and cardiac phenotypes (90).

Evidence for GLP-1RA is similarly conditional. A pooled analysis of semaglutide trials suggested a modest reduction in incident AF, but AF was not a primary endpoint in most contributing trials. Events were generally identified through adverse-event reporting, and rhythm-monitoring strategies were not standardized (91). In real-world analyses, GLP-1RA use was associated with a lower AF risk than dipeptidyl peptidase-4 inhibitor use, whereas no clear difference was observed in comparisons with SGLT2i (92). These findings illustrate how the choice of comparator therapy may influence observational estimates.

Other strategies that improve metabolic status have also been studied. Associations between metformin use and a lower risk of incident AF have been reported mainly in observational studies and may remain affected by diabetes severity, renal function, and prescribing patterns (93). From a non-pharmacological perspective, long-term studies of bariatric surgery suggest that substantial and sustained weight loss may be associated with a reduced risk of incident AF among individuals with severe obesity (94). Weight reduction, however, is typically accompanied by concurrent improvements in glycemic control, blood pressure, sleep apnea, and lipid status, making it difficult to identify the contribution of any single pathway.

The evidence for these metabolic therapies is derived predominantly from patients with diabetes, obesity, heart failure, or high cardiovascular risk and is therefore best interpreted as reflecting broader cardiometabolic treatment effects in risk-enriched populations. These findings cannot be unconditionally generalized to the wider population and do not establish changes in circulating lipids as the direct mediator through which treatment modifies incident AF risk.

#### 5.2.2 Treatment context and the interpretation of post-ablation recurrence evidence

Whereas studies of incident AF mainly examine how treatment exposure may influence long-term susceptibility to the arrhythmia, its interpretation is more complex after catheter ablation. In this setting, pharmacological and metabolic interventions act in patients with established AF and pre-existing atrial substrate abnormalities, and rhythm outcomes are additionally shaped by the ablation modality, lesion durability, recurrence definition, and intensity of rhythm monitoring. Treatment-related evidence should therefore be interpreted within the peri-ablation context rather than directly extrapolated from studies of incident AF.

Among lipid-lowering therapies, statins have been studied most extensively in relation to post-ablation recurrence. A network meta-analysis incorporating randomized trials suggested that statin therapy may be associated with a lower risk of recurrence (95). However, the evidence was derived predominantly from a limited number of relatively small studies involving RFCA, with substantial differences in statin dose, timing of initiation, treatment duration, and recurrence monitoring. The findings therefore suggest a possible benefit in selected patients but do not establish that any improvement in rhythm outcomes is mediated by LDL-C reduction or support a uniform peri-ablation statin regimen. Direct clinical evidence for PCSK9 inhibitors, ezetimibe, and fibrates remains scarce. Studies involving omega-3 fatty acids have also mainly evaluated observational associations between circulating fatty acid levels or ratios and recurrence rather than pharmacological intervention during the peri-ablation period.

More recent investigations have shifted toward comprehensive risk-factor management, weight reduction, and glucose-lowering therapy. In a randomized study using RFCA with pulmonary vein isolation as the procedural basis, comprehensive management of body weight, blood pressure, lipid levels, glycemic status, sleep apnea, and lifestyle factors improved post-ablation rhythm outcomes among overweight or obese patients with additional cardiometabolic risk factors (96). Because the intervention contained several components, however, the contribution of any individual change could not be determined.

A different randomized study, in which most patients underwent RFCA and a minority underwent cryoballoon ablation, found that structured weight management reduced BMI but did not produce a clear between-group difference in the primary AF burden endpoint (97). Comprehensive risk-factor management and isolated weight-loss intervention should therefore not be regarded as equivalent strategies. Their effects may depend on the magnitude and durability of weight reduction, intervention duration, AF phenotype, and whether other cardiometabolic risk factors improve concurrently.

Findings for metabolic medications appear particularly dependent on the population treated. In patients with diabetes undergoing catheter ablation, observational studies have associated SGLT2i use with a lower risk of recurrence (98). By contrast, a randomized study in patients without conventional indications for these agents—such as diabetes, heart failure, or chronic kidney disease—found that short-term dapagliflozin treatment did not reduce post-ablation AF burden or early recurrence (99). The difference between these findings may reflect variation in underlying disease, treatment indication, and the timing and duration of intervention rather than a direct contradiction between observational and randomized evidence.

Studies of GLP-1RA have mainly involved patients with obesity or diabetes. A meta-analysis suggested a possible reduction in post-ablation recurrence, but the contributing evidence was predominantly observational and showed substantial heterogeneity in patient characteristics, drug selection, degree of weight loss, and rhythm-monitoring strategies (100). In a propensity score–matched cohort of obese patients with paroxysmal AF undergoing RFCA, laser balloon ablation, or PFA, semaglutide use was accompanied by both substantial weight loss and a lower recurrence risk (101). Because treatment was not randomly assigned, the relative contributions of the medication, weight reduction, and patient selection cannot be separated.

A small randomized study of liraglutide in patients undergoing pulmonary vein isolation also reported a recurrence-reduction signal. However, the prespecified primary endpoint involving epicardial adipose tissue did not improve concurrently, and the rhythm finding should therefore be regarded as exploratory (102).

Similar difficulties in separating treatment effects from baseline patient differences apply to metformin. A retrospective cohort including patients treated with RFCA or cryoballoon ablation associated metformin use with a lower risk of recurrence (103). Patients not receiving metformin may, however, have had a greater prevalence of renal impairment or other contraindications to treatment, leaving the result susceptible to prescribing bias and residual confounding.

No current evidence demonstrates that improving a single lipid measure alone reduces AF recurrence after catheter ablation. Comprehensive risk-factor management may improve rhythm outcomes in some patients with a high cardiometabolic burden, whereas the potential effects of SGLT2i, GLP-1RA, and metformin remain strongly dependent on underlying disease and treatment indication. Any apparent benefit is therefore more plausibly interpreted as reflecting improvement in overall cardiometabolic status than as evidence for an independent effect mediated by changes in circulating lipid levels.

### 5.3 Clinical interpretation of lipid-related measures

At present, the available evidence is insufficient to translate circulating lipid measures or lipid-related composite indices into stand-alone prediction tools or direct determinants of treatment decisions. Their more realistic clinical role may be to provide additional information about a patient's overall risk profile. An abnormal lipid concentration or an elevated lipid-related index should not be interpreted as evidence that a particular lipid component directly causes incident AF or post-ablation recurrence. Instead, these measures should be considered alongside age, nutritional and frailty status, obesity, abnormalities in glucose metabolism, inflammatory burden, sleep apnea, heart failure, left atrial structural remodeling, EAT, treatment exposure, and the strategy used to detect recurrence. Lipid-related markers are therefore currently better viewed as peripheral indicators of the metabolic-inflammatory background and comorbidity burden than as isolated grounds for modifying clinical management.

A statistically independent association also does not necessarily translate into clinically meaningful incremental predictive value. Most existing studies have reported hazard ratios, odds ratios, or receiver operating characteristic curves for individual markers, but few have directly compared their performance with established clinical and imaging predictors. For most indices, external validation, model calibration, net reclassification improvement, integrated discrimination improvement, and decision-curve analysis remain unavailable. There is likewise no evidence that adding these measures to existing models changes the selection of ablation modality, postprocedural rhythm-monitoring strategies, or pharmacological management. Their present role should therefore be regarded as that of candidate risk markers rather than mature clinical prediction tools.

## 6 Conclusion

Current evidence does not demonstrate a stable and consistent direction of association between circulating lipids or lipid-related indices and either incident AF or recurrence after catheter ablation across populations and clinical stages. Conventional lipid measures, apolipoproteins, and composite indices related to insulin resistance are more likely to capture multidimensional information on metabolic burden, inflammatory status, comorbidity profiles, and the atrial substrate than to represent independent pathogenic or protective factors. At present, these markers may provide additional information for understanding a patient's overall risk context, but they are not sufficient for the stand-alone prediction of incident AF or post-ablation recurrence.

Future interventional studies should address more clinically relevant questions, including whether improving insulin resistance, controlling chronic inflammation, optimizing body weight and metabolic status, modifying lipid-lowering strategies, or strengthening peri-procedural lifestyle management can favorably alter the atrial substrate and reduce the risk of AF onset or recurrence after catheter ablation. The clinical value of lipid-related markers in AF risk stratification and post-ablation management will become clearer only when the current observational associations are extended through longitudinal monitoring, mechanistic validation, and evaluation of treatment-related benefits.
