# Supplementary material for: Circulating lipid profiles and atrial fibrillation: from incident risk to post-ablation recurrence
Source: Front Cardiovasc Med. 2026 Sep 2;13:1909014. doi: 10.3389/fcvm.2026.1909014 (PMC13579005; doi:10.3389/fcvm.2026.1909014)
Supplement: Supplementary file 1 [file Supplementaryfile1.docx]

| Ref. | Study | Full title | Study design and population | Ablation modality | Marker and exposure handling | Blanking period and recurrence endpoint | Rhythm monitoring | Follow-up | Recurrence events | Statistical model and key adjusted covariates | Main adjusted results and discrimination | Evidence role and key limitations |
| --- | --- | --- | --- | --- | --- | --- | --- | --- | --- | --- | --- | --- |
| 57 | Shang et al., 2020 | Blood lipid levels and recurrence of atrial fibrillation after radiofrequency catheter ablation: a prospective study | China; single center; Prospective observational cohort; 287 patients undergoing initial CPVA; paroxysmal and non-paroxysmal AF | RFCA; circumferential PVI using CARTO 3; additional treatment according to procedural findings | Fasting TC, LDL-C, HDL-C and TG before ablation; Quartiles and continuous analyses | Blanking period: 3 months. Endpoint: ECG/Holter-confirmed atrial tachyarrhythmia >30s after the blanking period | Visits every 3 months; 12-lead ECG and 48-h Holter even when asymptomatic; additional ECG for symptoms | 331 ± 163 days; outcome assessed during months 3–12 | 71/287 (24.7%) | Cox proportional-hazards models; Adjusted for: Sex, age, BMI, smoking, alcohol use, hypertension, diabetes, cardiovascular disease, LAD, AF type, ACEI/ARB, beta-blockers and lipid-lowering drugs | Lowest vs highest quartile: TC HR 3.66 (95% CI 1.56–8.56); LDL-C HR 2.28 (1.09–4.77). HDL-C and TG were not independently associated.; Associations were stronger in women; very large sex-specific HRs with wide CIs | Direct post-blanking recurrence cohort; Single center; small sex subgroups; residual confounding from treatment, diet and nutritional status |
| 58 | Gu et al., 2024 | Plasma triglyceride is associated with the recurrence of atrial fibrillation after radiofrequency catheter ablation: a retrospective study | China; single center; Retrospective cohort; 402 adults with paroxysmal or persistent AF undergoing first RFCA | RFCA; PVI with optional CTI or left-atrial linear ablation | Fasting pre-ablation TG; conventional lipids also recorded; Continuous TG and three categories: <1.28, 1.28–1.895, and >1.895 mmol/L | Blanking period: 3 months. Endpoint: AF/atrial tachyarrhythmia >30s occurring from 3 months to 1 year after ablation | Clinical review and 24-/48-h Holter; monthly ECG during the first 6 months; later telephone follow-up | Median 1.17 years | 79/402 (19.7%) | Multivariable Cox models; Adjusted for: Age, sex, BMI, platelet count, hypertension, heart failure and AF type | Per-unit TG HR 1.251 (1.019–1.536). Fully adjusted HRs were 2.331 (1.141–4.762) for moderate and 2.873 (1.332–6.199) for high TG vs low TG.; Dose-gradient across TG categories | Direct post-blanking recurrence cohort; Retrospective; changing procedural techniques; diet and repeated TG were not assessed |
| 59 | Tang et al., 2022 | The pre-ablation triglyceride-glucose index predicts late recurrence of atrial fibrillation after radiofrequency ablation in non-diabetic adults | China; single center; Retrospective cohort; 275 non-diabetic adults after exclusions; paroxysmal and persistent AF | RFCA; CARTO 3-guided circumferential PVI; additional trigger/CTI treatment when required | Fasting pre-ablation TyG; Continuous and ROC-derived cutoff analyses | Blanking period: 3 months. Endpoint: AF, atrial flutter or atrial tachycardia >30s after blanking | Visits at 1, 3 and 12 months, then annually; advised monthly ECG or 48-h Holter; symptom-triggered testing and telephone screening | Median 26.1 months | 70/275 (25.5%) late recurrence | Backward Cox regression; Adjusted for: Variables significant in univariable analysis and clinically established predictors; final model included TyG, age >65 years, LAD, early recurrence, APPLE score and DR-FLASH score | TyG HR 2.015 (1.408–4.117); TyG AUC 0.737; compared with APPLE and DR-FLASH scores | Direct post-blanking recurrence cohort; Non-diabetic population only; retrospective single center; intermittent monitoring may miss asymptomatic recurrence |
| 60 | Sang et al., 2025 | The Triglyceride-Glucose Index Predicts Early Recurrence of Atrial Fibrillation After Ablation in Non-Diabetic Patients | China; single center; Retrospective cohort; 417 non-diabetic patients undergoing first RFCA; paroxysmal or persistent AF | RFCA; ablation-index-guided CPVI with low-voltage substrate ablation when present | Fasting preoperative TyG; Continuous analysis and ROC assessment | Blanking period: Outcome was within the conventional 3-month blanking window. Endpoint: Early AF/atrial flutter/atrial tachycardia >30 s from 48 h to 3 months after RFCA | Continuous ECG for 48 h; monthly assessment, 12-lead ECG and 24-h Holter during months 1–3; additional monitoring for symptoms | 3 months for the primary endpoint | 117/417 (28.1%) early recurrence | Stepwise multivariable logistic regression; Adjusted for: Age, LAD and candidate variables significant in univariable analysis | TyG OR 3.608 (2.280–5.711); age OR 1.027 and LAD OR 1.101 also remained significant; TyG AUC 0.687; combination with age and LAD improved discrimination/reclassification | Early-recurrence study, not a post-blanking recurrence cohort; Retrospective single center; single TyG measurement; no gold-standard IR measure; intermittent outpatient monitoring |
| 61 | Wang et al., 2024 | Non-insulin-based insulin resistance indexes in predicting atrial fibrillation recurrence following ablation: a retrospective study | China; two centers; Retrospective cohort; 2,242 patients undergoing de novo RFCA; paroxysmal and persistent AF | RFCA; CPVI for all, with optional lines/CFAE/SVC treatment | Pre-ablation TyG, TyG-BMI, METS-IR and TG/HDL-C; Continuous, tertile and cutoff analyses | Blanking period: 3 months. Endpoint: AF/atrial tachyarrhythmia >30s on ECG/Holter after blanking within 1 year | Visits every 3 months during year 1; 12-lead ECG, 24-h Holter and symptom-triggered assessment | 1 year | 711/2,242 (31.7%) | Multivariable Cox models and incremental prediction analyses; Adjusted for: Age, sex, drinking, diabetes, hyperlipidemia, coronary disease, AF duration, AF type, HbA1c, LAD, platelet count and CHA2DS2-VASc; component variables excluded as appropriate | Top vs bottom tertile: METS-IR HR 1.82 (1.50–2.22); TyG-BMI HR 1.71 (1.41–2.07). TG/HDL-C was not consistently independent.; TyG-BMI provided the largest incremental predictive improvement among tested indices | Direct post-blanking recurrence cohort; Retrospective; residual confounding; one-year follow-up; intermittent monitoring; index-specific model differences |
| 62 | Qin et al., 2025 | Insulin resistance, left atrial anatomical remodeling, and recurrence in patients with atrial fibrillation undergoing radiofrequency ablation | China; single center; Retrospective observational cohort; 910 patients with non-valvular AF undergoing index RFCA | RFCA; PVI for all, with additional lines/substrate modification in non-paroxysmal AF | Fasting pre-ablation TyG-BMI and LAD; Continuous and tertile analyses; mediation analysis | Blanking period: 3 months. Endpoint: AF or atrial arrhythmia ≥30s after blanking | Follow-up at 3, 6 and 12 months via outpatient/readmission/telephone; 24-h Holter or 7-day extended ECG | Median 12 months (IQR 12–15) | 189/910 (20.8%) | Adjusted Cox, restricted cubic spline, ROC and mediation models; Adjusted for: Sex, AF type, AF duration, heart failure, BNP, HDL-C, hemoglobin and CHA2DS2-VASc | TyG-BMI continuous HR 1.015 (1.010–1.021); highest vs lowest tertile HR 3.026 (1.903–4.811); AUC: TyG-BMI 0.634, LAD 0.639; LAD mediated 19.23% of the association | Direct post-blanking recurrence cohort; Retrospective; LAD rather than LA volume/voltage; dynamic TyG-BMI not studied; additional ablation may confound; Chinese cohort |
| 63 | Jia et al., 2024 | Association between triglyceride-glucose index trajectories and radiofrequency ablation outcomes in patients with stage 3D atrial fibrillation | China; two centers; Retrospective cohort with latent-class trajectory modeling; 997 patients with stage 3D AF; paroxysmal and persistent AF | RFCA; PVI with optional additional linear ablation | TyG at baseline and during months 1–2 and 2–3 after RFCA; Baseline categories and latent trajectories (low-low, small intermediate class, high-high) | Blanking period: 3 months. Endpoint: AF/atrial flutter/atrial tachycardia ≥30s documented after blanking | 24-h ECG at 1, 3 and 6 months; 7-day ECG at 12 months; 24-h ECG every 6 months thereafter | 30.43 ± 17.75 months | 200/997 (20.1%) | Adjusted Cox models and latent-class mixed modeling; Adjusted for: Age, sex, BMI, AF duration/type, LAD, LVEF status and NT-proBNP | Baseline TyG HR 1.255 (1.087–1.448); high-high vs low-low trajectory HR 1.580 (1.146–2.177); Discrimination was modest: baseline TyG AUC 0.563; trajectory AUC 0.568 | Direct post-blanking recurrence and dynamic-exposure cohort; Retrospective; one trajectory class was small; residual confounding; intermittent rhythm monitoring |
| 64 | Yan et al., 2024 | Association between the cumulative triglyceride-glucose index and the recurrence of atrial fibrillation after radiofrequency catheter ablation | China; single center; Retrospective cohort; 375 patients completing follow-up after first RFCA; paroxysmal and persistent AF | RFCA; CPVI with optional additional lines | TyG measured at baseline, 1 month and 3 months; cumulative TyG calculated over time; Tertiles of cumulative TyG | Blanking period: 3 months. Endpoint: AF/atrial flutter/atrial tachycardia >30s after blanking | Visits at 1, 3, 6, 9 and 12 months; ECG and/or 24-h Holter; symptom-triggered testing | 1 year | 67/375 (17.9%) | Multivariable Cox models; Adjusted for: Age group, BMI >24, hyperlipidemia, persistent AF, AF duration >5 years, LDH, LAD, antiarrhythmic treatment, ablation protocol and cumulative TyG group | T2 vs T1 HR 4.949 (1.778–13.778); T3 vs T1 HR 8.716 (3.371–22.536); Cumulative TyG cutoff 2.11; combined model with LAD and LDH improved discrimination; AUC 0.845 in the main Results (0.847 in the abstract) | Direct post-blanking recurrence and cumulative-exposure cohort; Single-center retrospective design; one-year follow-up; limited covariates; 24-h Holter may miss asymptomatic recurrence |
| 66 | Zou et al., 2024 | L-shaped association of plasma low-density lipoprotein cholesterol with atrial fibrillation recurrence after catheter ablation: a prospective cohort study | China; single center; Prospective cohort; 482 patients undergoing de novo catheter ablation; predominantly paroxysmal AF | RFCA; PVI with additional lines/substrate modification when indicated | Fasting pre-procedural LDL-C; Quartiles, continuous analysis and threshold/spline modeling | Blanking period: 3 months. Endpoint: AF/atrial flutter/atrial tachycardia ≥30s after blanking | ECG/24-h Holter at 3, 6 and 12 months; symptom-triggered testing; telephone follow-up every 6 months after year 1 | Median 15 months (IQR 9–24) | 96/482 (19.9%) | Multivariable Cox and two-piecewise threshold models; Adjusted for: Extensive adjustment for demographics, AF characteristics, comorbidities, scores, thyroid markers, medications, inflammatory/metabolic markers and echocardiographic variables | LDL-C showed an L-shaped association. At LDL-C ≤3.20 mmol/L, each 1 mmol/L increase was associated with lower recurrence (HR 0.50, 0.33–0.74); above 3.20 mmol/L, HR 2.11 (0.76–5.89), not significant.; Nonlinear threshold analysis; fully adjusted continuous HR approximately 0.63–0.66 | Direct post-blanking recurrence cohort; Observational single center; few patients at high LDL-C; residual confounding and treatment effects |
| 67 | Li et al., 2023 | Association Between Pre-Radiofrequency Catheter Ablation Serum Lipid Levels and Recurrence of Atrial Fibrillation in 412 Patients in Beijing, China: A Single-Center Study | China; single center; Case-control study nested in an ablation cohort; 412 AF patients; paroxysmal and non-paroxysmal AF | RFCA; CPVI for paroxysmal AF and CPVI plus linear lesions for persistent AF | Fasting pre-ablation TG, HDL-C, LDL-C and TC; Quartiles with sex-stratified logistic models | Blanking period: 3 months. Endpoint: AF/atrial flutter/atrial tachycardia ≥30s after 3 months, confirmed by 12-lead ECG or 24-h Holter | 12-lead ECG or 24-h Holter; schedule and total follow-up duration were not clearly reported | Not reported | 82/412 (19.9%) | Sex-stratified multivariable logistic models; Adjusted for: BMI, diabetes, coronary disease, AF type, hypertension, LAD, lipid variables, CRP, TSH, fasting glucose, blood pressure and WBC | Overall continuous TC/LDL-C were not significant. Female-only quartile estimates were statistically unstable; TC Q3 vs Q4 HR 16.24 (1.14–231.56).; Published LDL-C female subgroup estimates contain an internally inconsistent HR/CI entry | Direct post-blanking recurrence study with substantial estimate instability; Single center; case-control design; follow-up schedule/duration not reported; small sex strata; very wide and internally inconsistent estimates |
| 68 | Li et al., 2020 | Association of clinical predictors with recurrence of atrial fibrillation after catheter ablation | China; single center; Retrospective cohort; 257 patients after RFCA; 174 paroxysmal and 83 persistent AF | RFCA; CARTO Merge-guided circumferential PVI | Pre-ablation LDL-C among a broad panel of clinical predictors; Continuous LDL-C | Blanking period: 3 months. Endpoint: AF/atrial tachycardia >30s after blanking | Visits at 1, 3 and 6 months, then every 6 months; 12-lead ECG and 24-h Holter; 24–72-h Holter for symptoms | 11.63 ± 3.64 months (range 6–24) | 79/257 (30.7%) | Multivariable logistic regression; | LDL-C OR 1.646 (1.129–2.398); AF duration and pulmonary arterial hypertension were also independent predictors; LDL-C sensitivity 60.5% and specificity 60.6% | Direct post-blanking recurrence cohort; Retrospective single center; modest sample; some follow-up as short as 6 months; intermittent monitoring |
| 69 | Canpolat et al., 2015 | The role of preprocedural monocyte-to-high-density lipoprotein ratio in prediction of atrial fibrillation recurrence after cryoballoon-based catheter ablation | Türkiye; single center; Prospective observational cohort; 402 patients undergoing cryoballoon PVI; 325 paroxysmal and 77 persistent AF | First-generation cryoballoon PVI | Fasting pre-procedural MHR; Quartiles and continuous Cox analysis | Blanking period: 3 months. Endpoint: Documented AF ≥30s after blanking | Visits at 3, 6 and 12 months and every 6 months thereafter; 24-h Holter at scheduled visits and earlier for symptoms | 20.6 ± 6.0 months | 95/402 (23.6%) | Multivariable Cox regression; Adjusted for: Age, BMI, AF type/duration, symptom class, LAD, WBC, MHR and early recurrence | MHR HR 1.20 (1.15–1.25); Cutoff 11.48; sensitivity 85%, specificity 74% | Direct post-blanking recurrence cohort; Single-center observational study; single MHR measurement; no direct oxidative-stress biomarker assessment |
| 70 | Chen et al., 2020 | The preablation monocyte/high density lipoprotein ratio predicts the late recurrence of paroxysmal atrial fibrillation after radiofrequency ablation | China; single center; Retrospective cohort; 125 patients with paroxysmal AF | RFCA; circumferential PVI with optional roof-line ablation | MHR from blood collected 24 h before RFCA; Continuous and tertile analyses | Blanking period: 3 months. Endpoint: AF/atrial tachycardia >30s after blanking | Visits at 3, 6 and 12 months and every 6 months; 48-h Holter or 12-lead ECG; symptom-triggered review | 25.1 ± 12.0 months | 47/125 (37.6%) | Backward multivariable logistic regression; Adjusted for: AF duration, diabetes, BMI, monocyte count, HDL-C, MHR and LAD entered from univariable screening | MHR OR 1.34 (1.12–1.60); LAD OR 1.21 (1.08–1.35); AUC: MHR 0.712; LAD 0.739 | Direct post-blanking recurrence cohort; Retrospective single center; small paroxysmal-only cohort; intermittent monitoring |
| 71 | Lei and Hu, 2024 | The predictive value of monocyte count to high-density lipoprotein cholesterol ratio combined with left atrial diameter for post-radiofrequency ablation recurrence of paroxysmal atrial fibrillation in patients | China; single center; Observational cohort; 210 patients with first-onset paroxysmal AF after exclusions | First RFCA; procedural technical details were not fully described in the main text | Preoperative MHR and LAD; Continuous Cox analysis; median cutoffs MHR 33.58 and LAD 36.05 mm | Blanking period: Recurrence was defined from 3 months after ablation; a formal blanking statement was not separately provided. Endpoint: AF >30s on routine ECG or dynamic ECG from 3 months after RFCA | Monthly review with history, examination, ECG and echocardiography; 24-h dynamic ECG when necessary | 12 months | 78/210 (37.1%) | Cox regression; Adjusted for: Diabetes, hypertension, AF onset time, hs-CRP, MHR and LAD | MHR effect estimate 1.104 (1.055–1.155); LAD effect estimate 1.028 (1.014–1.042); hs-CRP and AF onset time also significant; AUC: MHR 0.827; LAD 0.809; combined 0.894 | Direct recurrence cohort; Single center; one-year follow-up; unusually high diabetes prevalence; procedural details limited; terminology occasionally inconsistent |
| 72 | Song et al., 2024 | Significance of the APPLE score and the monocyte–HDL cholesterol ratio in predicting late recurrence of atrial fibrillation following catheter ablation based on a retrospective study | China; single center; Retrospective cohort; 438 patients; 303 paroxysmal and 135 persistent/long-standing persistent AF | RFCA in 291 and cryoballoon ablation in 147 | Pre-ablation MHR and APPLE score; Continuous analysis and combined score | Blanking period: 3 months. Endpoint: AF/atrial flutter/atrial tachycardia >30s on ECG/Holter after 3 months and off antiarrhythmic drugs | Regular visits/telephone; 72-h ambulatory ECG at 3, 6 and 12 months; symptom-triggered ECG | Median 11 months (IQR 8–17) | 115/438 (26.3%) late recurrence; 91/438 (20.8%) early recurrence | Multivariable Cox regression; Adjusted for: Age, early recurrence, HDL-C, MHR, APPLE score, LAD, hyperthyroidism history and AF duration entered after univariable screening | MHR HR 1.061 (1.005–1.121); APPLE HR 1.566 (1.237–1.982); early recurrence HR 3.424 (2.356–4.976); AUC: MHR 0.643; APPLE 0.653; combined 0.702; similar performance across RFCA/CBA subgroups | Direct post-blanking recurrence cohort; Retrospective; selection/follow-up bias; 72-h monitoring still misses asymptomatic episodes; combined score lacks external validation |
| 73 | Dervis et al., 2024 | The Uric Acid-to-High-Density Lipoprotein Cholesterol Ratio: A New Biomarker for Predicting Arrhythmia Recurrence After Atrial Fibrillation Ablation | Türkiye; two centers; Retrospective cohort; 163 patients with paroxysmal, persistent or long-persistent AF; repeat ablation excluded | RFCA in 71 and cryoballoon ablation in 92 | Pre-ablation UHR; uric acid and UCR also analyzed; Median split and ROC-derived cutoff >15.1 | Blanking period: Text states a 1-month blanking period, but also classifies 0–3 months as early and 3–12 months as late recurrence. Endpoint: AF or atrial flutter detected during 1-year follow-up; analyses combined recurrence categories | Clinical assessment and ECG at 1, 3 and 12 months; 24-h Holter for symptoms | 1 year | 39/163 (23.9%) | Three multivariable logistic models to avoid collinearity; Adjusted for: LAD, long-persistent AF and CRP, with uric acid/UHR/UCR entered in separate models | UHR >15.1 OR 3.824 (1.682–8.693); LAD and long-persistent AF also remained significant; UHR AUC 0.665 (0.562–0.768) | Direct recurrence cohort, but endpoint-window terminology is internally inconsistent; Small retrospective cohort; symptom-driven Holter; blanking-period inconsistency; no external validation |
| 74 | Yang et al., 2025 | Serum uric acid to high-density lipoprotein cholesterol ratio: a novel biomarker for predicting atrial fibrillation recurrence after cryoablation | China; single center; Retrospective cohort; 519 symptomatic drug-refractory non-valvular AF patients; paroxysmal or persistent AF | Second-generation cryoballoon PVI | Pre-ablation UHR; repeated UHR at 3, 6 and 12 months and up to 36 months; Continuous and quartile analyses; ROC cutoff 413.46 in the article's reported units | Blanking period: 90 days. Endpoint: AF/atrial flutter/atrial tachycardia ≥30s after blanking; late recurrence 3–12 months and extremely late recurrence >12 months | Continuous ECG for 24 h post-procedure; ECG and 24-h Holter before discharge and every 3 months; symptom-triggered ECG | 36 months | 120/519 (23.1%) | Multivariable logistic regression and time-to-event analyses; Adjusted for: Male sex, hypertension, BMI, LAD, AF type and other significant variables | Reported effect estimates (labeled as HR in the original article): continuous UHR 1.016 (1.012–1.019); persistent AF 2.993 (1.394–6.426); AUC 0.848 for any recurrence, 0.880 for late recurrence and 0.803 for extremely late recurrence; cutoff 413.46 | Direct long-term recurrence cohort; Single center; unusual UHR scale/cutoff; very large quartile effects; retrospective design; no external validation; the original report uses logistic regression but labels the effect estimates as HR. |
| 75 | Wu et al., 2023 | Predictive value of remnant-like particle cholesterol in the prediction of long-term AF recurrence after radiofrequency catheter ablation | China; single center; Retrospective cohort; 320 patients with AF undergoing successful first RFCA; paroxysmal and persistent AF | RFCA; CPVI, with additional lines for persistent AF or induced arrhythmias | Fasting pre-ablation calculated RLP-C = TC − LDL-C − HDL-C; Continuous per 0.1 mmol/L and cutoff 0.645 mmol/L | Blanking period: 3 months. Endpoint: Atrial tachyarrhythmia >30s on ECG or 24-h Holter after 3 months | First visit 7 days after discharge, then every 3 months; routine 12-lead ECG and additional 24-h Holter; symptom-triggered review/telephone | 1 year | 103/320 (32.2%) | Backward multivariable Cox regression; Adjusted for: Age, sex, BMI, diabetes, AF type, early recurrence, coronary disease, LAD, LVEF, TC, TG, LDL-C, HDL-C and RLP-C | RLP-C HR 0.75 per 0.1 mmol/L increase (0.68–0.83); >0.645 vs ≤0.645 mmol/L HR 0.21 (0.11–0.39); AUC 0.81; sensitivity 87.4%, specificity 63.6% | Direct post-blanking recurrence cohort; Retrospective single center; calculated rather than direct RLP-C; lipid-lowering drug users excluded; no repeated lipid measurements |
| 77 | Yang Xuesong, 2024 | Correlation between apolipoprotein B/apolipoprotein A1 ratio and recurrence of atrial fibrillation after radiofrequency catheter ablation | China; single center master’s thesis; Retrospective cohort; 232 drug-refractory AF patients undergoing successful first RFCA | CARTO 3-guided circumferential PVI | Preoperative ApoB/ApoA1 ratio; Continuous Cox analysis and cutoff 0.855 | Blanking period: Not explicitly specified. Endpoint: Recurrent AF, atrial tachycardia or atrial flutter >30s during follow-up | ECG/Holter before discharge and at 3 months, then twice yearly; telephone every 6 months; symptom-triggered ECG | At least 12 months for non-recurrent patients; | 60/232 (25.9%) | Multivariable Cox regression; Adjusted for: AF type, ApoB/ApoA1, LAD and D-dimer in the reported multivariable table | ApoB/ApoA1 HR 2.868 (1.230–6.688); LAD HR 1.064; D-dimer HR 1.364; ApoB/ApoA1 AUC 0.611 (0.533–0.689); cutoff 0.855 | Direct recurrence cohort, lower evidence tier because it is a thesis; Single center; thesis/non-peer-reviewed evidence; blanking period not stated; modest discrimination; potential treatment-selection bias; The narrative text and the reported multivariable table are internally inconsistent regarding D-dimer. |
| 78 | Zuin et al., 2025 | Lipoprotein(a) and risk of recurrent atrial fibrillation after pulsed field ablation | Italy; single center; Retrospective observational cohort; 133 consecutive PFA patients with baseline Lp(a); mixed AF types | Pulsed-field ablation | Fasting baseline Lp(a); Percentile groups and restricted cubic spline | Blanking period:3 months. Endpoint: Recorded AF >30s after blanking | Outpatient visits, medical records and hospitalization records; a standardized ECG/Holter schedule was not reported | Median 7.8 months after blanking (range 6.4–9.3) | 29/133 (21.8%) | Adjusted Cox and restricted cubic spline models; Adjusted for: Age, BMI, hypertension, AF type, CHA2DS2-VA, diabetes and left atrial volume | Adjusted HRs increased across Lp(a) percentiles: 1.11, 1.22 and 1.28 vs the lowest half; risk became significant above approximately 46 mg/dL; Dose-response spline; preliminary PFA-specific association | Direct post-blanking recurrence cohort; Small single-center Caucasian cohort; short follow-up; no standardized monitoring schedule; preliminary findings |
| 79 | Duan et al., 2025 | Multimodal data-based longitudinal prognostic model for predicting atrial fibrillation recurrence after catheter ablation in patients with patent foramen ovale and paroxysmal atrial fibrillation | China; single center; Retrospective prognostic-model cohort with development/validation split; 145 patients with PFO and new-onset paroxysmal AF; development n=102, validation n=43 | Catheter ablation; the energy source was not clearly reported in the main article, which refers to supplemental methods | Pre-ablation Lp(a) within 41 multimodal candidate variables; LASSO selection followed by backward Cox modeling | Blanking period: 3 months. Endpoint: Documented AF/atrial flutter/atrial tachycardia ≥30s after blanking | 24-h Holter at 3, 6, 9 and 12 months; biannual 12-lead ECG and 24-h Holter thereafter; additional ECG for symptoms | Median 36 months | 44/145 (30.3%) | LASSO and backward multivariable Cox; nomogram development and internal split-sample validation; Adjusted for: Final model included age, diabetes, Lp(a) and right ventricular diameter | Lp(a) HR 1.002 per reported unit (1.001–1.004); Nomogram AUCs at 1/2/3 years: 0.911/0.812/0.786 in development and 0.842/0.761/0.785 in validation | Special-population prognostic model; not a general Lp(a)-focused cohort; Small highly selected PFO+PAF sample; internal rather than external validation; modality not explicit in main text; possible overfitting |

**Supplementary Table S1. Characteristics of original clinical studies evaluating lipid or lipid-related markers in relation to atrial fibrillation recurrence after catheter ablation**

Scope note: This table includes original clinical studies that directly evaluated lipid or lipid-related markers with post-ablation AF or atrial tachyarrhythmia recurrence as the outcome. The systematic review and meta-analysis [65] is synthesized in the main text and is not listed as an original cohort in this table.

Evidence-boundary note: Sang et al. [60] evaluated early recurrence within the conventional blanking period; Yang Xuesong [77] is a master's thesis; Duan et al. [79] is a prognostic-model study in the special population of patients with PFO and paroxysmal AF.

Exclusion note: Bielawiec et al. [76] evaluated only acute peri-cryoablation changes in apolipoproteins and sphingolipids and did not analyze AF recurrence as the statistical outcome; it was therefore excluded from this recurrence-study table. Reference numbers are consistent with the latest manuscript.

***Abbreviations:*** ACEI, angiotensin-converting enzyme inhibitor; AF, atrial fibrillation; ApoA1, apolipoprotein A1; ApoB, apolipoprotein B; ARB, angiotensin receptor blocker; BMI, body mass index; CBA, cryoballoon ablation; CPVI, circumferential pulmonary vein isolation; ECG, electrocardiogram; HDL-C, high-density lipoprotein cholesterol; LAD, left atrial diameter; LDL-C, low-density lipoprotein cholesterol; Lp(a), lipoprotein(a); METS-IR, metabolic score for insulin resistance; MHR, monocyte-to-HDL-C ratio; PFA, pulsed-field ablation; PFO, patent foramen ovale; PVI, pulmonary vein isolation; RFCA, radiofrequency catheter ablation; RLP-C, remnant-like particle cholesterol; TC, total cholesterol; TG, triglyceride; TyG, triglyceride-glucose index; TyG-BMI, triglyceride-glucose index–body mass index; UHR, uric acid-to-HDL-C ratio.
